# *Sclerotinia sclerotiorum* utilizes host-derived copper for ROS detoxification and infection

**DOI:** 10.1371/journal.ppat.1008919

**Published:** 2020-10-01

**Authors:** Yijuan Ding, Jiaqin Mei, Yaru Chai, Wenjing Yang, Yi Mao, Baoqin Yan, Yang Yu, Joseph Onwusemu Disi, Kusum Rana, Jiana Li, Wei Qian

**Affiliations:** 1 College of Agronomy and Biotechnology, Southwest University, China; 2 Academy of Agricultural Sciences, Southwest University, China; 3 Key Laboratory of Plant Molecular Physiology, Institute of Botany, Chinese Academy of Sciences, China; 4 College of Plant Protection, Southwest University, China; 5 Department of Entomology, University of Georgia, Athens, United States of America; Institute of Microbiology, CHINA

## Abstract

Necrotrophic plant pathogen induces host reactive oxygen species (ROS) production, which leads to necrosis in the host, allowing the pathogen to absorb nutrients from the dead tissues. *Sclerotinia sclerotiorum* is a typical necrotrophic pathogen that causes Sclerotinia stem rot in more than 400 species, resulting in serious economic losses. Here, we found that three *S*. *sclerotiorum* genes involved in copper ion import/transport, *SsCTR1*, *SsCCS* and *SsATX1*, were significantly up-regulated during infection of *Brassica oleracea*. Function analysis revealed that these genes involved in fungal ROS detoxification and virulence. On the host side, four genes putatively involved in copper ion homeostasis, *BolCCS*, *BolCCH*, *BolMT2A* and *BolDRT112*, were significantly down-regulated in susceptible *B*. *oleracea*, but stably expressed in resistant *B*. *oleracea* during infection. Their homologs were found to promote resistance to *S*. *sclerotiorum* and increase antioxidant activity in *Arabidopsis thaliana*. Furthermore, copper concentration analysis indicated that copper flow from healthy area into the necrotic area during infection. A model was proposed that *S*. *sclerotiorum* utilizes host copper to detoxify ROS in its cells, whereas the resistant hosts may restrict the supply of essential copper nutrients to *S*. *sclerotiorum* by maintaining copper ion homeostasis during infection.

## Author summary

*Sclerotinia sclerotiorum* is a devastating necrotrophic fungal pathogen and has a substantial economic impact on crop production worldwide. *S*. *sclerotiorum* successfully infect the host plants by promote ROS production in host and induce plant necrosis. This raises an interesting question of how this pathogen survive from high level of host-derived ROS. Copper is an essential nutrient for microbial pathogens and serves as an important cofactor for enzymes of scavenging ROS. In our previous study, we simultaneously analyzed the transcriptome changes of *Brassica oleracea* and *S*. *sclerotiorum* during inoculation. The results in present study show that *S*. *sclerotiorum* induces the genes involved in ‘copper ion import’ and ‘copper ion transport’ during infection to promote the ROS detoxification and fungal virulence. While, the resistance host maintains the genes involved in ‘copper ion homeostasis’, which promote the resistance to *S*. *sclerotiorum* and antioxidant activity. Furthermore, copper concentration is higher in the necrotic area than the margin and healthy area, which may indicate that copper flow from healthy area into the necrotic area during infection. In summary, our results highlight a more subtle and complex role of copper during the interaction of *S*. *sclerotiorum* and its host plants.

## Introduction

Copper serves as a cofactor in many enzymes and is an essential micronutrient for growth and development of organisms. It is involved in a range of biological processes, including photosynthetic and respiratory electron transport, cell wall remodeling, oxidative stress responses, and ethylene perception [1, 2]. Given its importance, copper homeostasis has been well-studied in mammalian, bacteria, yeast model systems and the filamentous fungi [3]. In yeast, Cu^2+^ is reduced to Cu^+^ by cell membrane metalloreductases (Fre1 and Fre2), and Cu^+^ is then transported into cells by the high-affinity Cu^+^ transporters Ctr1 and Ctr3 [4]. The Ctr2 transporter mobilizes the stored copper from the vacuole into the cytosol under low-copper conditions [5]. The cytosolic copper is delivered to the cuproenzymes in diverse ways. For example, the copper homeostasis factor Atx1 binds and delivers copper into Fet3 via the Ccc2 pump in yeast [6, 7]. Copper chaperone CCS delivers copper into Cu/Zn SOD in human and yeast [8, 9], and copper is transferred into cytochrome *c* oxidase in the mitochondria via copper chaperones such as COX17 and COX11 in eukaryotes [10, 11]. A few genes related to the absorption and distribution of copper have been discovered in *Arabidopsis thaliana*, such as genes encoding copper transporters (COPTs), chaperone components (CCH, CCS and COX), metallothioneins (MTs), P-type ATPases (HMA, PAA and RAN) and plastocyanin (PETE) [12–14].

Reactive oxygen species (ROS) including hydrogen peroxide (H_2_O_2_), hydroxyl radical (HO-), singlet oxygen (^1^O_2_) and superoxide anion (·O_2_^-^), are derived from partial reduction of oxygen (O_2_) [15]. ROS have been called ‘double-edged swords of life’ [16]. On the one hand, ROS act as signaling molecules that regulate development, differentiation, redox levels, stress signaling, interactions with other organisms and systemic responses [17]. On the other hand, excess ROS cause oxidative cellular injury to DNA, RNA, proteins and lipids, and also trigger programmed cell death [16, 18, 19]. To avoid or overcome the damage caused by excess of ROS, organisms have developed a complex ROS scavenging system that delicately regulates the balance between production and elimination of ROS. A few enzymes, such as the superoxide dismutase (SODs), ascorbateperoxidase (APX), glutathione S-transferase (GST) and tripeptide glutathione (GSH) are involved in ROS scavenging and antioxidant activity. Among them, Cu/Zn superoxide dismutase (Cu/Zn SOD) constitutes the front-line defense against ROS, which dismutase the ·O_2_^-^ to hydrogen peroxide [20]. Copper homeostasis factor ATX1 act as a multicopy suppressor of oxygen toxicity in cells lacking Cu/Zn SOD [21], while cytochrome *c* oxidase catalyzes the reduction of oxygen to water in mitochondria [22].

Necrotrophic plant pathogens promote ROS production in the plant host and induce necrosis during host colonization [23]. This raises an interesting question of how necrotrophic plant pathogens survive in such high levels of host-derived ROS. *Sclerotinia sclerotiorum* is a typical necrotrophic pathogen that causes Sclerotinia stem rot in more than 400 species, including a few important crops such as rapeseed, soybean, resulting in serious production losses [24]. In this study, our data showed that *S*. *sclerotiorum* enhanced the expression of genes involved in copper ion import and transport in order to utilize the host-derived copper to activate ROS detoxification enzymes during infection, and that resistant hosts may limit the supply of copper to *S*. *sclerotiorum* by maintaining copper ion homeostasis. This research provides new insights into the interaction between *S*. *sclerotiorum* and the host, highlighting the subtle and complex role of copper in these interactions.

## Results

### Copper is involved in the interaction between *Brassica oleracea* and *S*. *sclerotiorum*

**Fig 1:**
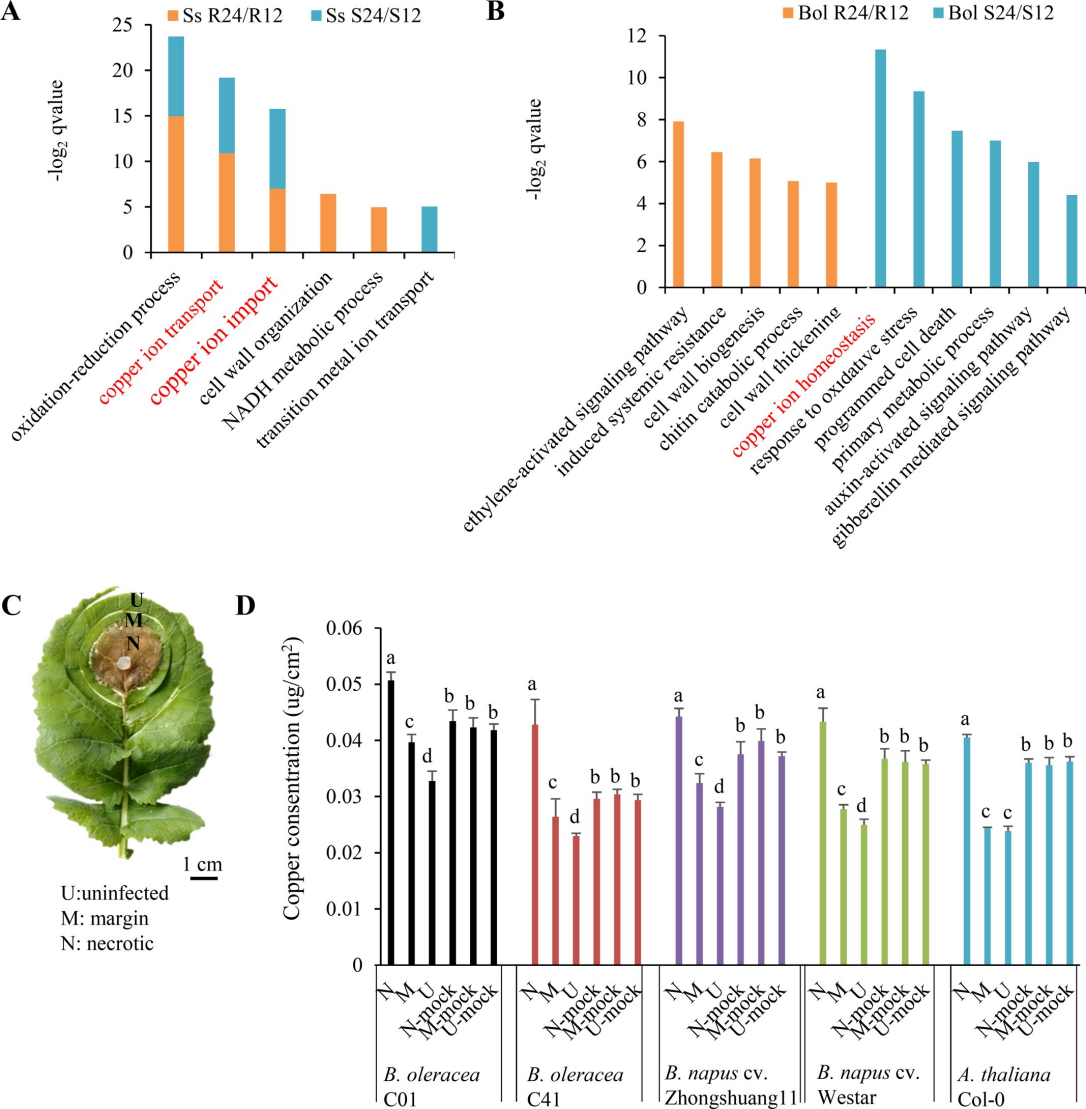
Copper is involved in the interaction between *Brassica oleracea* and *Sclerotinia sclerotiorum*. (A) Gene Ontology (GO) biological processes represented by *S*. *sclerotiorum* DEGs (differentially expressed genes; 24 hours post inoculation [hpi] vs 12 hpi) in infected *B*. *oleracea*. (B) GO biological processes represented by resistant and susceptible *B*. *oleracea* DEGs (24 hpi vs 12 hpi). Ss R24/R12: *S*. *sclerotiorum* DEGs in resistant *B*. *oleracea* identified by comparing 24 hpi to 12 hpi; Ss S24/S12: *S*. *sclerotiorum* DEGs in susceptible *B*. *oleracea* identified by comparing 24 hpi to 12 hpi; Bol R24/R12: *B*. *oleracea* DEGs in resistant *B*. *oleracea* identified by comparing 24 hpi to 12 hpi; Bol S24/S12: *B*. *oleracea* DEGs in susceptible *B*. *oleracea* identified by comparing 24 hpi to 12 hpi. (C) Schematic workflow of tissue sampling in copper content analysis. (D) Copper concentration in uninfected (U), margin (M), necrotic (N) and mock tissues of resistant parental *B*. *oleracea* C01, susceptible parental *B*. *oleracea* C41, resistant *B*. *napus* cv. Zhongshuang 11 and susceptible *B*. *napus* cv. Westar leaves at 48 hpi and in *A*. *thaliana* wild-type Col-0 at 12 hpi. Error bars indicate the standard deviation of three independent replicates with six leaves for every sample in one replicate. Different letters indicate statistically significant differences (*P* < 0.05, Student's *t*-test).

*S*. *sclerotiorum* induces typical lesions, which are the main battlegrounds of gene interactions between *S*. *sclerotiorum* and the host. We previously detected differentially expressed genes (DEGs) by comparing gene expression in lesions of resistant and susceptible F_2_ plants of *B*. *oleracea* [25]. Here, the set of transcriptome data was analyzed for dynamic changes of gene expression in *S*. *sclerotiorum* and hosts during infection. A total of 738 and 228 *S*. *sclerotiorum* DEGs (24 hours post inoculation [hpi] vs 12 hpi) were detected in lesions of resistant and susceptible *B*. *oleracea*, respectively (S1A Fig), which were significantly enriched for three overlapping Gene Ontology (GO) terms, ‘oxidation–reduction process’, ‘copper ion transport’ and ‘copper ion import’ (Fig 1A). Eight *S*. *sclerotiorum* DEGs involved in the ‘copper ion transport’ and ‘copper ion import’ processes were up-regulated during infection as revealed by both RNA-seq analysis and quantitative real-time reverse transcription-polymerase chain reaction (qRT-PCR) analysis (S1B and S2A Figs).

A total of 5988 and 5441 DEGs (24 hpi vs 12 hpi) were detected and subjected to GO analysis in resistant and susceptible *B*. *oleracea*, respectively (S1C Fig). Interestingly, the biological process ‘copper ion homeostasis’ was significantly enriched in susceptible *B*. *oleracea* but not in resistant *B*. *oleracea* (Fig 1B). Among ten DEGs involved in ‘copper ion homeostasis’ (S1D Fig), seven genes (*Bol023613*, *Bol026950*, *Bol044257*, *Bol002542*, *Bol011307*, *Bol000591* and *Bol029708*) with consistent expression patterns between RNA-seq analysis and qRT-PCR analysis were significantly down-regulated in susceptible *B*. *oleracea* plants, but only slightly down-regulated or stably expressed in resistant *B*. *oleracea* plants (S2B Fig). We further analyzed their expression in parental resistant (C01) and susceptible (C41) *B*. *oleracea* lines via qRT-PCR. All seven genes showed sharply down-regulated expression in susceptible parental line C41, while six of the seven genes (*Bol023613*, *Bol044257*, *Bol002542*, *Bol011307*, *Bol000591* and *Bol029708*) showed a stably or even slightly up-regulated expression in the resistant parental line C01 (24 hpi vs 12 hpi) (S2C Fig). This suggests that copper ion homeostasis is disrupted in susceptible *B*. *oleracea* during early infection.

### Copper distribution in and around lesions during early infection

Considering that the ‘copper ion transport’ and ‘copper ion import’ processes are promoted in *S*. *sclerotiorum* during infection, we speculate *S*. *sclerotiorum* absorbs copper from lesion for infection, producing copper flows from the healthy area into the necrotic area in host. We analyzed the copper distribution in the different sites of healthy leaves, and in and around lesions of infected leaves in several hosts, including parental *B*. *oleracea* (C01 and C41) and two cultivars in *B*. *napus* (cv. Zhongshuang 11 and Westar) at 48 hpi and *A*. *thaliana* (Col-0) at 12 hpi. Although the copper concentration exhibit significant differences in the healthy leaves among five hosts (*P* < 0.05, 0.043 ± 0.001 μg/cm^2^ in *B*. *oleracea* C01, 0.030 ± 0.001μg/cm^2^ in *B*. *oleracea* C41, 0.038 ± 0.001μg/cm^2^ in *B*. *napus* cv. Zhongshuang11, 0.036 ± 0.001μg/cm^2^ in *B*. *napus* cv. Westar and 0.036 ± 0.001μg/cm^2^ in *A*. *thaliana* Col-0), no significant difference for the copper concentration was detected in the different sites of healthy leaves of each host (Fig 1D). However, the copper concentration exhibited significant difference among the necrotic, margin and uninfected areas of leaves in each host (*P* < 0.05) (Fig 1D). The copper concentration in necrotic areas was 27.86% and 62.08% higher than that of margin areas, and 54.61% and 86.32% higher than that of uninfected areas in parental *B*. *oleracea* C01 and C41, respectively (Fig 1D). Similar results that highest copper concentration in necrotic areas were detected in *B*. *napus* cv. Zhongshuang11 and Westar and *A*. *thaliana* Col-0 (Fig 1D). These data indicate that copper flows from healthy area into the necrotic area during early infection.

### Copper ion homeostasis genes promote host resistance

**Fig 2:**
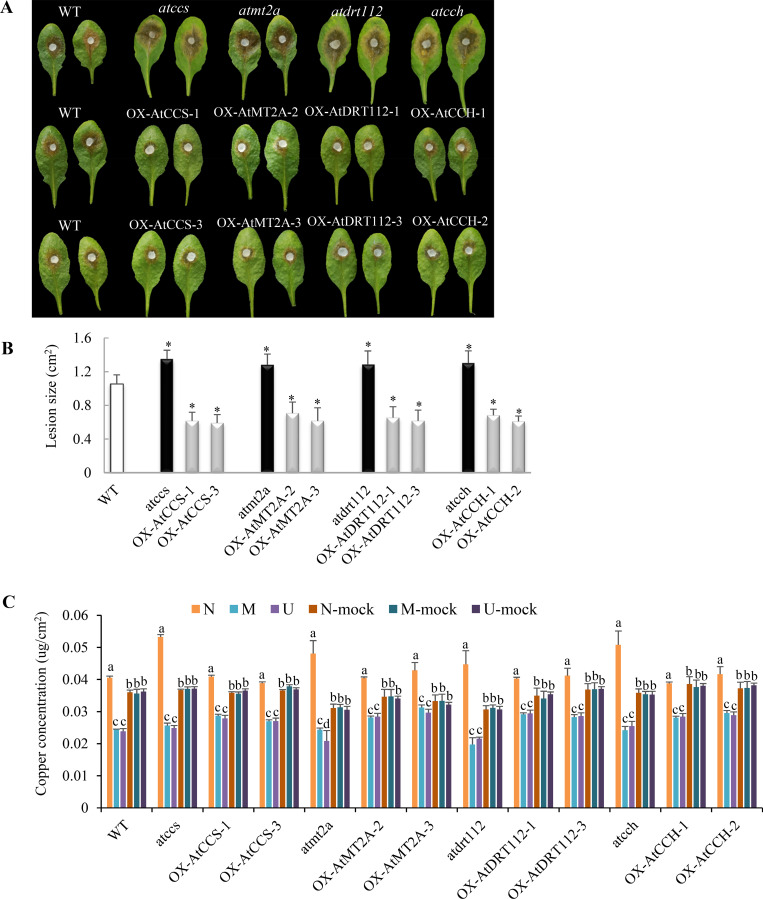
Copper ion homeostasis genes promote resistance to *S*. *sclerotiorum* in *Arabidopsis thaliana*. (A) Disease symptoms in *A*. *thaliana* wild-type line Col-0 (WT), T-DNA mutants (*atccs*, *atmt2a*, *atdrt112* and *atcch*) and overexpression lines (OX-AtCCS, OX-AtMT2A, OX-AtDRT112 and OX-AtCCH) corresponding to copper ion homeostasis genes at 24 hpi with *S*. *sclerotiorum* wild-type strain 1980. (B) Quantitation of Lesion sizes in (A). Error bars indicate the standard deviation of six independent replicates with five leaves for every sample in one replicate. *: represents significant difference from WT at 0.05 level (Student's *t*-test). (C) Copper concentration in uninfected (U), margin (M), necrotic (N) and mock tissues of *A*. *thaliana* WT, T-DNA mutants and overexpression at 12 hpi. Error bars indicate the standard deviation of three independent replicates with twenty leaves for every sample in one replicate. Different letters indicate statistically significant differences (*P* < 0.05, Student's *t*-test).

To test whether copper ion homeostasis is associated with host resistance, the six genes in this process that were stably expressed or slightly up-regulated in resistant *B*. *oleracea* but significantly down-regulated in susceptible *B*. *oleracea* were aligned with four *A*. *thaliana* orthologs (*AtCCS*, *AtMT2A*, *AtDRT112* and *AtCCH*) (S3A Fig). We tested the function of these *A*. *thaliana* homologs with respect to *S*. *sclerotiorum* resistance using T-DNA mutants (*atccs*, *atmt2a*, *atdrt112* and *atcch*) and overexpression lines (OX-AtCCS, OX-AtMT2A, OX-AtDRT112 and OX-AtCCH) (S3B and S3C Fig). Notably, all of the reduced-expression T-DNA mutants were more susceptible to *S*. *sclerotiorum* compared to the wild-type line, while the overexpression lines displayed higher resistance (Fig 2A). At 24 hpi, the lesion size was 1.28–1.35 cm^2^ in the T-DNA mutant lines, 1.06 cm^2^ in the wild-type line and 0.59–0.71 cm^2^ in the overexpression lines (Fig 2B). More interesting, the necrotic areas of the T-DNA mutants showed the highest copper concentration, followed by that of wild-type line and overexpression lines of four *A*. *thaliana* genes (Fig 2C).

### Copper ion homeostasis is associated with response to oxidative stress in the host

To further explore how copper ion homeostasis is associated with host resistance, the transcriptomes of leaves from *A*. *thaliana* wild-type line, T-DNA mutants and overexpression lines of *AtCCS*, *AtMT2A*, *AtDRT112* and *AtCCH* at 0, 6, and 12 hpi were sequenced, producing an average of 22.9 million clean reads for each sample. On average, 17.71 and 0.97 million clean reads were mapped to the reference genome of *A*. *thaliana* and *S*. *sclerotiorum* per sample, respectively. As expected, the overexpression lines exhibited higher expression of the corresponding target genes than the T-DNA mutants and the wild-type line (S4A Fig). Five common GO terms, including ‘response to oxidative stress’, were detected by comparing the up-regulated DEGs between overexpression lines and T-DNA mutants and between overexpression lines and wild-type line at 12 hpi (S4B Fig). Considering that more DEGs were found between the overexpression lines and T-DNA mutants than between the overexpression lines and wild-type line, we conducted a Weighted Gene Co-expression Network Analysis (WGCNA) using a total of 7321 *A*. *thaliana* DEGs between the overexpression lines and T-DNA mutants (S1 Table). This analysis produced 17 modules (groups of genes with similar expression pattern), of which one (shown in red) showed a negative correlation with lesion size (*r* = -0.45, *P* = 0.03) (S5A Fig). It encompassed 394 genes, which were significantly enriched for the biological process of ‘response to oxidative stress’ (S5B Fig). In addition, total of 1273 *S*. *sclerotiorum* DEGs (overexpression lines vs. T-DNA mutants) were detected and used for WGCNA, which resulted in five significant modules (*P* < 0.05), two of which were highly correlated with the processes of ‘peroxisome organization’ and ‘oxidation–reduction’ (S2 Table and S5C Fig).

**Fig 3:**
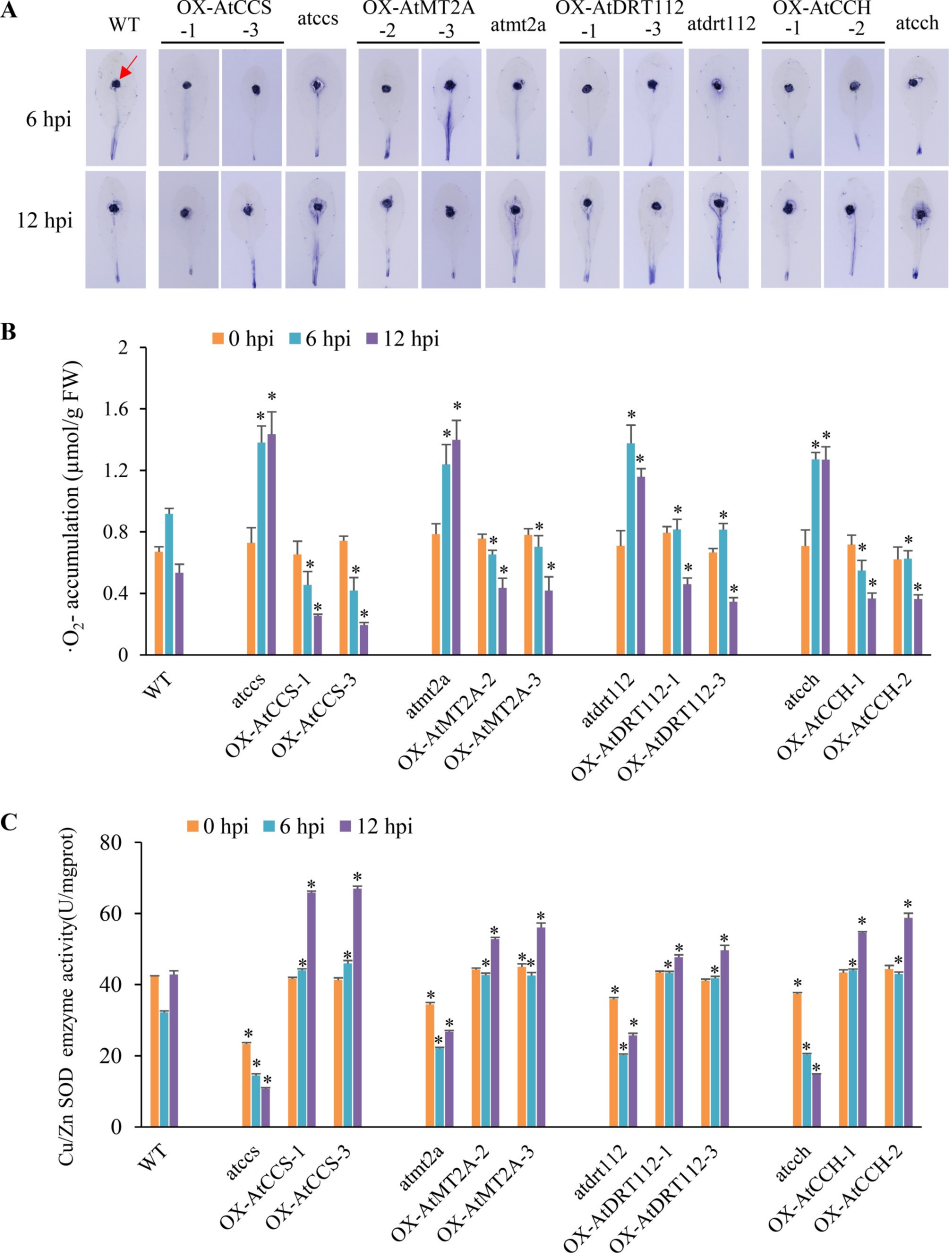
Copper ion homeostasis genes promote antioxidant activity in host. Antioxidant activity assays in wild-type line Col-0 (WT), T-DNA mutants and overexpression lines (OX) of copper ion homeostasis genes in *A*. *thaliana* at 0, 6 and 12 hpi. (A) NBT (·O_2_^-^ accumulation) staining. One representative replicate from the five independent experiments is shown. Three leaves were stained in each experiment. The red arrow indicate the inoculant column. (B) Quantitative of ·O_2_^-^ accumulation in (A). (C) Enzyme activity of Cu/Zn SOD in *A*. *thaliana* leaves at 0, 6 and 12 hpi. Error bars indicate standard deviation for three independent replicates with three leaves for every sample in one replicate. *: represents significant difference from WT at 0.05 level (Student's *t*-test).

The oxidative burst is a typical response of a host against pathogen attack [26]. To functionally test whether copper ion homeostasis genes are associated with response to oxidative stress, we analyzed the antioxidant activity of these overexpression lines and T-DNA mutants by staining inoculated leaves at 0, 6 and 12 hpi with nitrotetrazolium blue chloride (NBT) for the accumulation of ·O_2_^-^. We observed deeply stained areas around the inoculant column and more accumulation of ·O_2_^-^ (1.32 ± 0.07 μmol/g FW at 6 hpi, and 1.31 ± 0.13 μmol/g FW at 12 hpi) in the T-DNA mutants, but more lightly stained areas and less accumulation of ·O_2_^-^ in the overexpression lines (0.63 ± 0.15 μmol/g FW at 6 hpi, and 0.35 ± 0.09 μmol/g FW at 12 hpi) (Fig 3A and 3B), suggesting that antioxidant activity was enhanced in the overexpression lines but suppressed in the T-DNA mutants. This conclusion was further supported by a Cu/Zn SOD enzyme activity assay, in which the overexpression lines (43.30 ± 1.26 and 56.53 ± 7.02 U/mgprot at 6 and 12 hpi, respectively) exhibited higher Cu/Zn SOD enzyme activity than T-DNA mutants (19.26 ± 3.32 and 19.48 ± 8.00 U/mgprot at 6 and 12 hpi, respectively) (Fig 3C). Wild-type line was in the middle (32.14 and 42.82 U/mgprot at 6 and 12 hpi, respectively). These results indicate that copper ion homeostasis is associated with the detoxification of ROS in the host.

### *S*. *sclerotiorum* requires trace copper for pathogen infection

**Fig 4:**
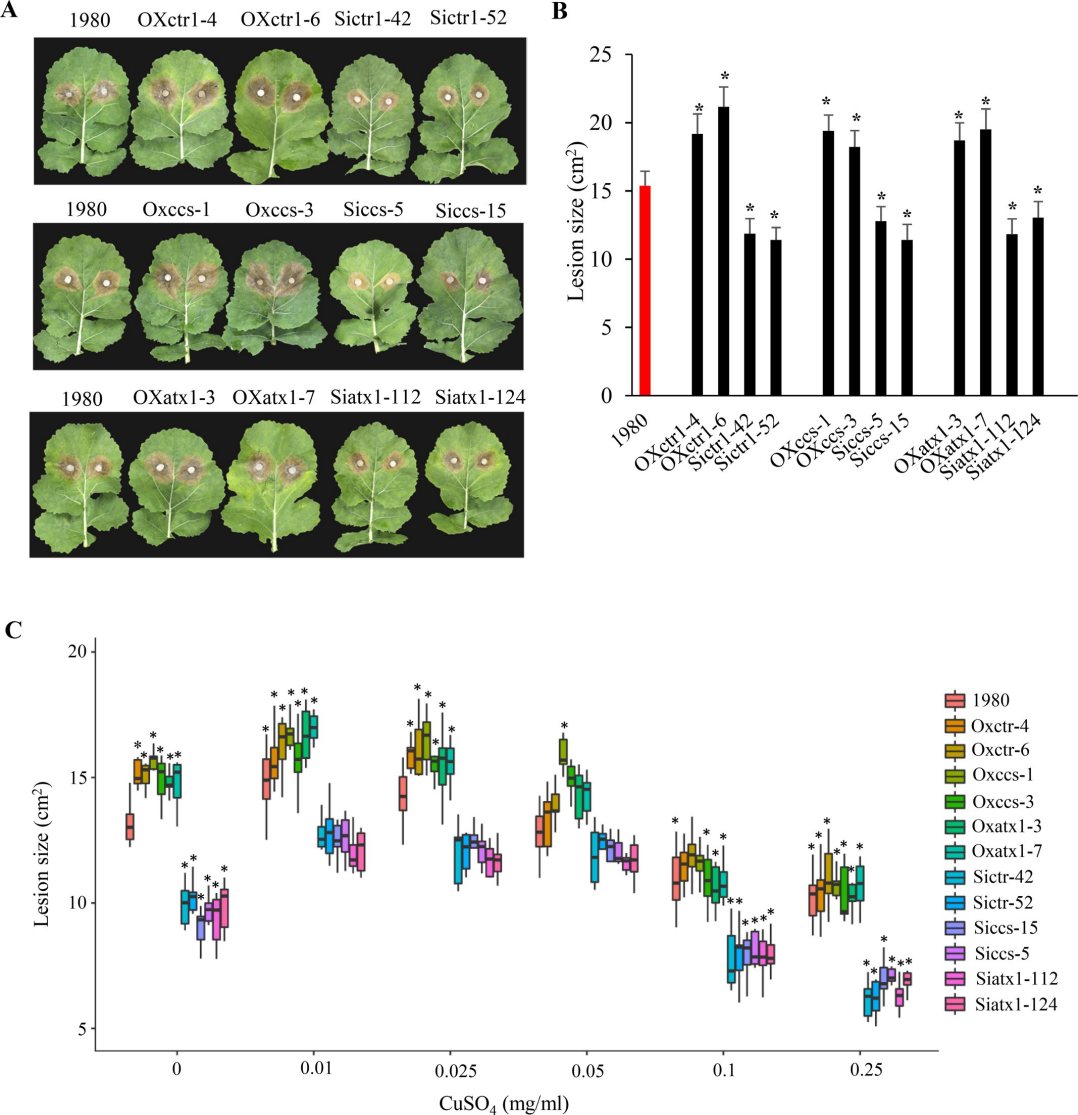
Trace copper restores the virulence of silenced strains of *S*. *sclerotiorum*. (A) Disease symptoms of *B*. *napus* cv. Zhongshuang 11 leaves at 48 hpi with *S*. *sclerotiorum* wild-type strain 1980, overexpression strains (OX) and silenced strains (Si) for genes involved in ‘copper ion transport/import’. One representative replicate from the five experiments is shown. Five leaves were analyzed for each strain in each experiment. (B) Quantitation of lesion sizes at 48 hpi in (A). *: represents significant difference from wild-type strain at 0.05 level (Student's *t*-test). (C) Comparison of the lesion sizes on *B*. *napus* cv. Zhongshuang 11 leaves produced by wild-type strain, silenced and overexpression strains supplemented with low- and high-concentration of CuSO_4_ solutions at 48 hpi. Error bars indicate standard deviation of five independent replicates with five leaves for every sample in one replicate. *: represents significant difference from wild-type strain 1980 which sprayed with ddH_2_O (0 mg/L CuSO_4_ solutions) at 0.05 level (Student's *t*-test).

Copper is an essential nutrient for microbial pathogens and serves as an important cofactor of enzymes that scavenge ROS [8, 10]. To test the hypothesis that *S*. *sclerotiorum* takes up and utilizes host-derived copper for the synthesis or activation of enzymes involved in ROS scavenging during infection, we explored the roles of three *S*. *sclerotiorum* DEGs annotated as involved in ‘copper ion transport/import’: *SS1G_05578* (*SsCTR1*), *SS1G_00102* (*SsCCS*) and *SS1G_10888* (*SsATX1*) with their silenced and overexpression strains (S6A and S6B Fig). The products of these genes function to transport extracellular copper into the fungal cell (*SsCTR1*), deliver copper to Cu/Zn SOD (*SsCCS*) and detoxify the oxidative damage (*SsATX1*) [21, 27, 28]. The lesion size after inoculation with the silenced strains was smaller than those after inoculation with the wild-type strain, while the largest lesion size was observed after inoculation with the overexpression strains in the detached leaves of *B*. *napus*, T-DNA mutants, overexpression lines and wild-type line of *A*. *thaliana* seedlings (Fig 4A and 4B, S6C and S6D Fig), indicating that these genes are involved in the virulence of *S*. *sclerotiorum*.

To test the role of copper in the virulence of *S*. *sclerotiorum*, we sprayed low-concentration CuSO_4_ solutions (0.01, 0.025 and 0.05 mg/L) onto the leaf surface and incubated for 30 min prior to inoculation with each of the silenced strains. The lesion size after inoculation with the silenced strains increased by 1.19- to 1.35-fold at 48 hpi in comparison with the non-CuSO_4_ treatment (sprayed with ddH_2_O), but exhibited no significant difference from that inoculated with the wild-type strain and sprayed with ddH_2_O (Fig 4C). However, treatment sprayed with highly concentrated CuSO_4_ solutions (0.1 and 0.25 mg/L) significantly reduced disease symptoms of the silenced strains (Fig 4C). Similar observations were detected in the wild-type strain and overexpression stains, which increased lesion size under low concentration of CuSO_4_, but decreased lesion size under high concentration of CuSO_4_ (Fig 4C). We further found that low-concentration CuSO_4_ promoted the growth of all the strains on potato dextrose agar (PDA) plates, and that high-concentration CuSO_4_ suppressed the growth of *S*. *sclerotiorum* (S7 Fig). Especially, the growth inhibition was higher in the silenced strains than the overexpression strains under high-concentration CuSO_4_ (0.25 mg/L) (S7 Fig). It suggests that low-concentration copper could promote the growth of strains and restores the virulence of the silenced strains.

**Fig 5:**
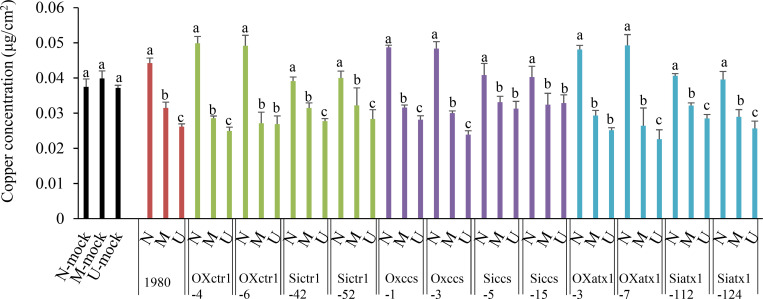
*SsCTR1*, *SsCCS* and *SsATX1* associates with copper absorption during infection. Copper concentration in uninfected (U), margin (M) and necrotic (N) tissues of *B*. *napus* cv. Zhongshuang11 leaves at 48 hpi with wild-type strain 1980, silenced and overexpression strains. Error bars indicate the standard deviation of three independent replicates with six leaves for every sample in one replicate. Different letters indicate statistically significant differences (*P* < 0.05, Student's *t*-test).

Furthermore, the copper concentration in the leaves infected by the silenced and overexpression strains of these three genes was determined (Fig 5). Similarly, the necrotic areas exhibited the higher copper concentration than the uninfected and margin areas of leaves. The copper concentration in the necrotic areas infected by the wild-type strain was 8.37%-13.09% higher than that of the silenced strains, and was 7.99%-11.27% lower than that of the overexpression strains (Fig 5). It suggests that *SsCTR1*, *SsCCS* and *SsATX1* are in association with copper uptake in *S*. *sclerotiorum* during infection.

### Copper ion transport/import involves in ROS scavenging in *S*. *sclerotiorum*

**Fig 6:**
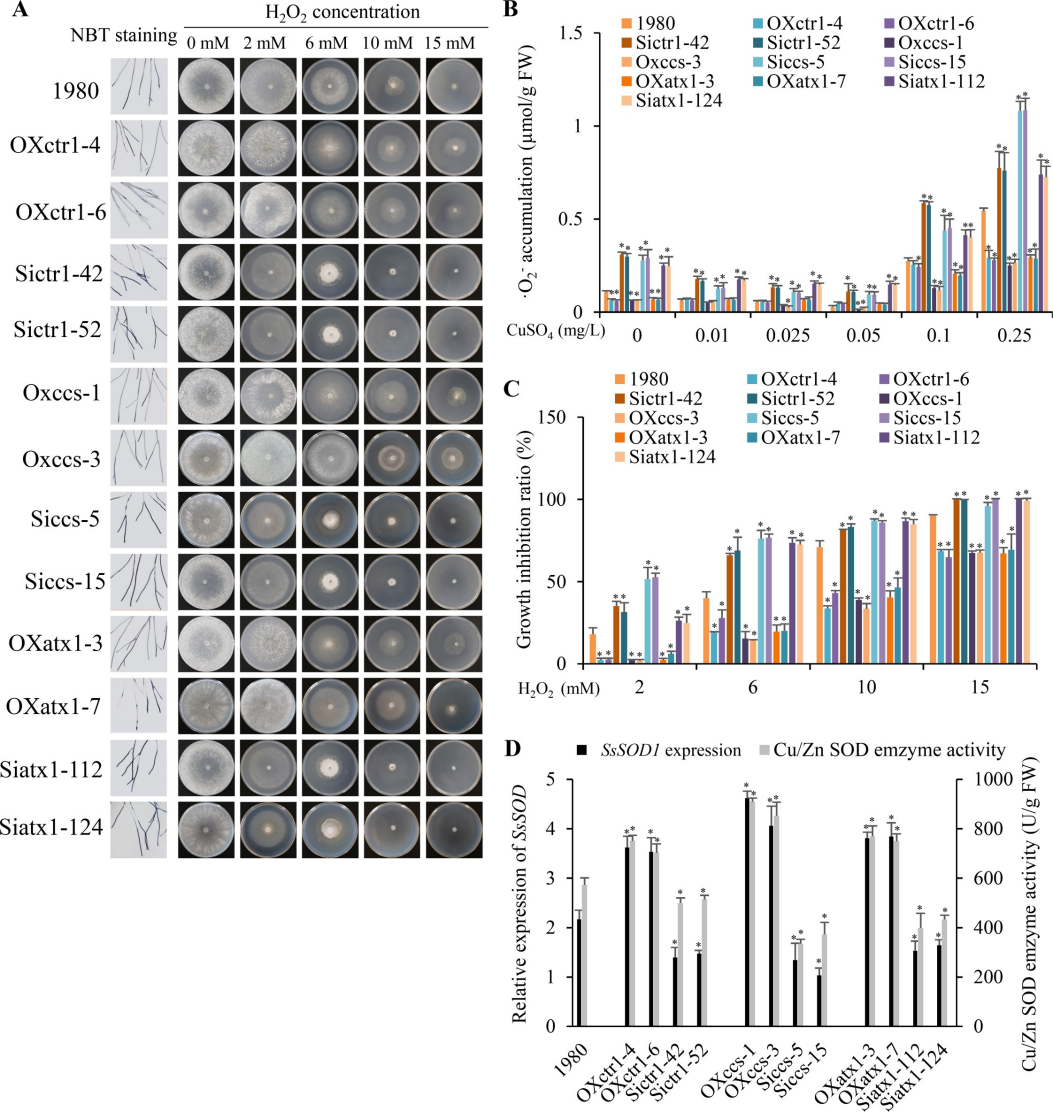
*SsCTR1*, *SsCCS* and *SsATX1* promotes antioxidant activity in *S*. *sclerotiorum*. (A) Accumulation of ·O_2_^-^ (NBT staining at 24 hpi) in the hyphae tips and growth phenotypes on PDA supplemented with different concentrations of H_2_O_2_ at 4 dpi. Five biological replicates in every independent experiments were analyzed. One representative replicate from the five experiments is shown. (B) Quantitative of ·O_2_^-^ accumulation when supplemented with low- and high-concentration of CuSO_4_ solutions. (C) The inhibition rate of hyphal growth on PDA supplemented with different concentrations of H_2_O_2_. In (B) and (C), error bars indicate standard deviation of five independent experiments with five plates for every sample in one replicate. (D) Relative expression of *S*. *sclerotiorum SsSOD1* and enzyme activity of Cu/Zn SOD in wild-type strain 1980, silenced and overexpression strains. Relative expression of *S*. *sclerotiorum SsSOD1* was indicated by qRT-PCR analysis. The quantity of *S*. *sclerotiorum SsTubulin* cDNA was used to normalize different samples. Error bars indicate the standard deviation of three independent samples. *: represents significant difference from wild-type strain 1980 at 0.05 level (Student's *t*-test).

To further test the hypothesis that *S*. *sclerotiorum* utilizes copper to promote ROS scavenging, we observed superoxide (·O_2_^-^) accumulation among wild-type strain, the silenced and overexpression strains of *SsCTR1*, *SsCCS* and *SsATX1* using NBT staining. The accumulation of ·O_2_^-^ in the fungal hyphal tips was highest in the silenced strains, followed by the wild-type strain and the overexpression strains (Fig 6A and 6B). We further found that exogenous spraying of low-concentration CuSO_4_ solutions (0.01, 0.025 and 0.05 mg/L) decreased the accumulation of ·O_2_^-^ but exogenous spraying of high-concentration CuSO_4_ solutions (0.1 and 0.25 mg/L) increased the accumulation of ·O_2_^-^ in all strains (Fig 6B). We also calculated the accumulation of ·O_2_^-^ in wild-type line, T-DNA mutants and overexpression lines of four *A*. *thaliana* genes which were infected with wild-type strain, silenced and overexpression strains of three *S*. *sclerotiorum* genes at 12 hpi, and found the highest accumulation of ·O_2_^-^ in the *A*. *thaliana* mutants infected with *S*. *sclerotiorum* overexpression strains and the lowest accumulation of ·O_2_^-^ in the *A*. *thaliana* overexpression lines infected with silenced strains (S8 Fig). We then cultured all strains on PDA supplemented with different concentrations of H_2_O_2_ (0, 2, 6, 10 and 15 mM) (Fig 6A). The growth of the silenced strains was most seriously inhibited (reduced by 24.80–52.56% at 2 mM H_2_O_2_, 66.04%-76.66% at 6 mM H_2_O_2_, 81.48%-86.79% at 10 mM H_2_O_2_ and 95.86%-100.00% at 15 mM H_2_O_2_), followed by the wild-type strain (reduced by 18.00% at 2 mM H_2_O_2_, 39.92% at 6 mM H_2_O_2_, 70.95% at 10 mM H_2_O_2_ and 90.26% at 15 mM H_2_O_2_) and the overexpression strains (reduced by 1.83–5.99% at 2 mM H_2_O_2_, 14.14%-27.85% at 6 mM H_2_O_2_, 33.41%-46.42% at 10 mM H_2_O_2_ and 64.75%-69.37% at 15 mM H_2_O_2_) (Fig 6C). These observations indicate that ROS can inhibit the growth of *S*. *sclerotiorum* and that these *S*. *sclerotiorum* genes are involved in the detoxification of ROS.

Because Cu/Zn SOD is one of the primary superoxide scavengers [16], we analyzed the *SsSOD1* expression level in the wild-type strain, silenced and overexpression strains. The *SsSOD1* transcript accumulation of wild-type strain was 1.51, 1.86 and 1.37 times of silenced strains, and 0.61, 0.50 and 0.57 times of overexpression strains of *SsCTR1*, *SsCCS* and *SsATX1* on average, respectively (Fig 6D). In comparison with the wild-type strain (572.51 U/g FW), the Cu/Zn SOD enzyme activity was lower in silenced strains of *SsCTR1*, *SsCCS* and *SsATX1* with average of 506.71, 353.51 and 415.23 U/g FW, and higher in their overexpression strains with average of 726.84, 881.30 and 759.57 U/g FW, respectively (Fig 6D). These results indicate that these three *S*. *sclerotiorum* copper-related genes promote the expression of *SsSOD1* and increase enzyme activity of Cu/Zn SOD, resulting in the increase of ROS scavenging capacity in the fungal cells.

## Discussion

Copper is a component of numerous enzymes and plays a key role in the responses to oxidative stress [20, 29]. By analyzing DEGs at lesion for dynamic changes in host and *S*. *sclerotiorum* during infection, we found that the genes in the process of ‘copper ion import’ and ‘copper ion transport’ with up-regulated expression involved in *S*. *sclerotiorum* copper uptake, virulence and ROS detoxification, and that the host genes in the process of ‘copper ion homeostasis’ with stable expression in the resistant line, but down-regulated expression in the susceptible line were associated with response to oxidative stress and resistance to *S*. *sclerotiorum*. Our data indicate a copper battlefield at the host-*S*. *sclerotiorum* interface where *S*. *sclerotiorum* utilizes host-derived copper to successfully detoxify ROS and colonize the host.

The generation of ROS at the infection site is one of the earliest responses of pathogen-associated molecular-pattern-triggered immunity (PTI) [26]. As secondary messengers, ROS are indispensable for signaling, stress responses and developmental processes [30]. However, excess ROS trigger programmed cell death (PCD) and cause host necrosis, which facilitates the growth of necrotrophic pathogens [23, 31, 32]. Antioxidant components in the host, such as peroxidase, SODs, glutathione sulfhydryl transferase (GST) and glutathione (GSH) are associated with resistance against *S*. *sclerotiorum* [33–38], and resistance against *S*. *sclerotiorum* can be improved by decreasing the accumulation and/or production of ROS in the host [39, 40]. The four DEGs in the biological process of ‘copper ion homeostasis’ we detected during infection are involved in various antioxidant activities. *AtCCS* is responsible for the activation of Cu/Zn SOD [41]. Metallothioneins (MTs), which act as heavy metal chelators and ROS scavengers, contribute to plant adaptation to abiotic stresses [42]. *DRT112* is one of two *A*. *thaliana* plastocyanin genes (*PETE2*), which function to buffer excess copper [14]. *AtCCH* is a homolog of yeast *ATX1*, which functions to deliver copper into laccase or Fet3 [21]. In this study, we found that the overexpression of these genes in *A*. *thaliana* enhanced host ROS detoxification and resistance against *S*. *sclerotiorum*, and the genes in the biological process of ‘copper ion homeostasis’ were coordinately expressed with those for ‘response to oxidative stress’. Therefore, our research provides evidence that copper ion homeostasis is associated with ROS detoxification in host.

Free copper ions induce ROS production [43], which may be toxic to *S*. *sclerotiorum*. We found that highly concentrated CuSO_4_ solutions suppressed the growth of *S*. *sclerotiorum*. In fact, copper is one of important active ingredients in many bactericidal and fungicidal agents, such as Bordeaux mixture [44]. However, *S*. *sclerotiorum* can grow in necrotic areas with a relatively high concentration of copper. Three aspects of our findings might help explain this phenomenon. (1) The copper uptake system of *S*. *sclerotiorum* was elevated during infection. *SsCTR1*, which functions to import extracellular copper into the fungal cells [45], exhibited up-regulated expression, indicating that host-derived copper may be imported into the *S*. *sclerotiorum* cells. (2) A few genes associated with copper detoxification, such as MTs (*SsMT*), Sur7 (*SsSUR7*) and P-type ATPase (*SsATP7A*) [46–48], were not significantly induced in *S*. *sclerotiorum* during infection (S9 Fig), indicating that the amount of host-derived copper was not high enough to produce toxic effects in *S*. *sclerotiorum*. (3) *SsCCS* and *SsATX1* expression was up-regulated during infection. The homologs of *SsCCS* function to deliver copper to oxidative scavengers Cu/Zn SOD [9, 27]. *SsSOD1* is identified as an important virulence factor of *S*. *sclerotiorum* and plays critical roles in detoxification of ROS during host–pathogen interactions and is an important virulence factor of *S*. *sclerotiorum* [49, 50]. The ATX1 gene could act as a multi-copy suppressor of oxidative damage in yeast cell lacking SOD1 [21]. It indicates that the copper might be utilized for the synthesis or activation of these ROS detoxification enzymes of *S*. *sclerotiorum* during infection. Thus, this study provides important insights into the question about survival of *S*. *sclerotiorum* at relatively high levels of ROS.

**Fig 7:**
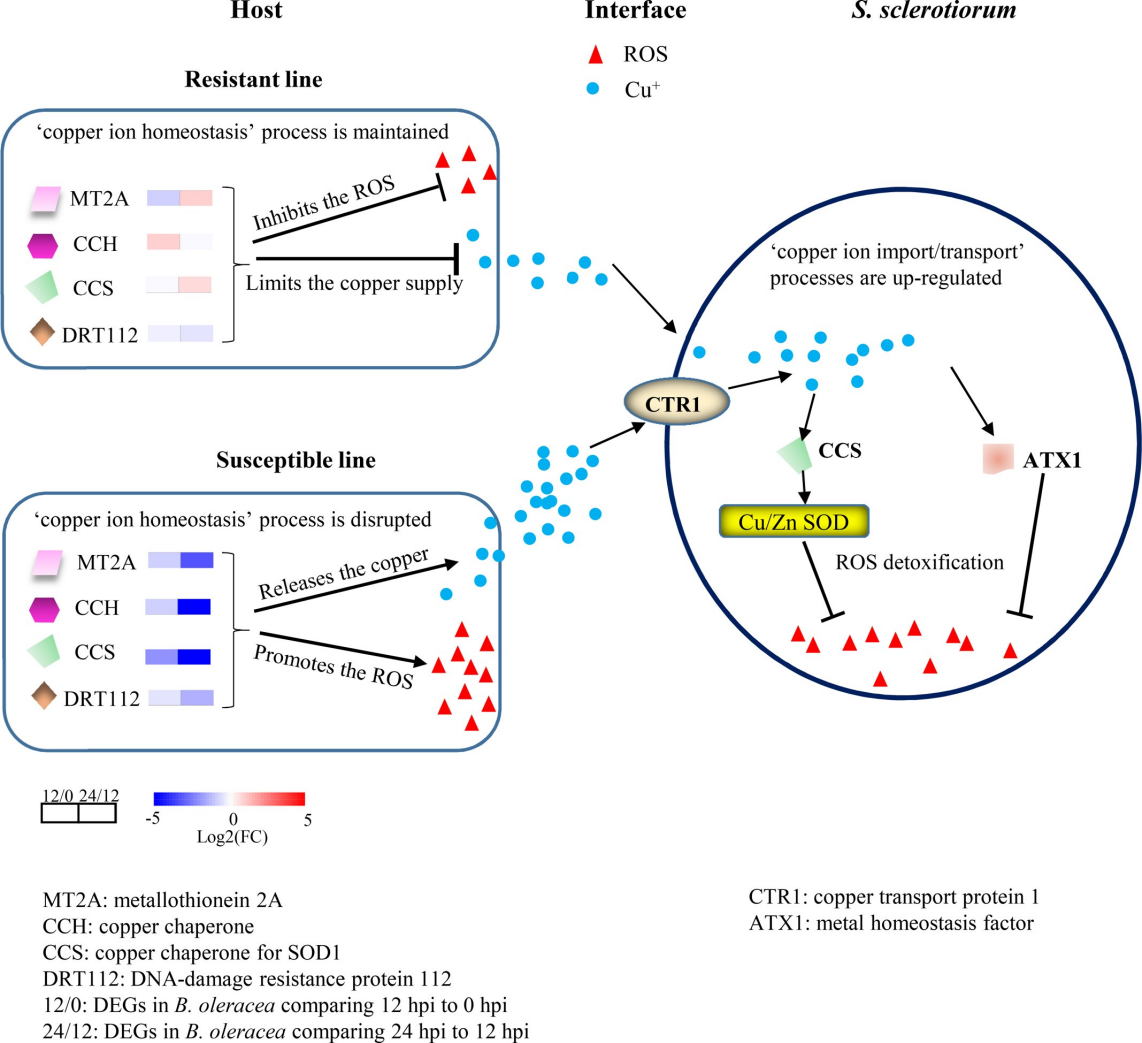
Model depicting the battle for copper acquisition between host and *S*. *sclerotiorum* during infection. *S*. *sclerotiorum* promotes its copper uptake systems to acquire host-derived copper, and activates its ROS scavenging system for survival and virulence, while a resistant host induces ‘nutritional immunity’ that restricts the supply of essential copper nutrients to *S*. *sclerotiorum* by maintaining ‘copper ion homeostasis’. 12/0: gene expression comparison of 12 hpi to 0 hpi in host, 24/12: gene expression comparison of 24 hpi to 12 hpi in host.

The idea of ‘nutritional immunity’ was first proposed to describe the resistance mechanism in human cells wherein they withhold transition metals, such as iron, zinc, manganese and copper to defend against microbial pathogen invaders [51–54]. To overcome this strategy, successful pathogenic species must evolve specialized mechanisms to adapt to the nutritionally restrictive environment of the host and cause disease. For example, the human kidney and brain can thwart *Candida albicans* growth by limiting the supply of copper nutrients, producing ‘copper starvation’ to *C*. *albicans* [51]. In response, *C*. *albicans* induces copper uptake machineries that enable it to survive in a copper-starved environment [55]. Here, the defense response in resistant lines that stabilized the expression of copper ion homeostasis genes and limited the availability of copper to *S*. *sclerotiorum* may be considered a form of nutritional immunity. To the best of our knowledge, this is the first description of nutritional immunity in plants. We therefore propose a possible host–*S*. *sclerotiorum* interaction model in which resistant plants induce nutritional immunity and restrict the supply of essential copper nutrients to *S*. *sclerotiorum* by maintaining copper ion homeostasis, while *S*. *sclerotiorum* enhances its copper uptake system and acquires host-derived copper, which activates its ROS scavenging system during infection and promotes its survival and virulence (Fig 7).

## Methods and materials

### Experimental strains and plants

The wild-type strain of *S*. *sclerotiorum* 1980 [56] were used in this study. Fungal strains were grown on potato dextrose agar (PDA, 20% potato, 2% dextrose and 1.5% agar) at 22°C. *S*. *sclerotiorum* transformants were cultured on PDA amended with 80 μg/ml hygromycin B (Calbiochem, San Diego, CA) to stabilize the transformants. Seedlings from *A*. *thaliana* Col-0 (ecotype Columbia-0), T-DNA mutants and overexpression transgenic lines were grown in the autoclaved soil (Pindstrup) at 20 ± 2°C under a 12 h light/dark cycle with 70% relative humidity.

### Vector construction and transformation of *S*. *sclerotiorum* and *A*. *thaliana*

The fragments of three *S*. *sclerotiorum* genes (*SsATX1*: 221 bp, *SsCCS*: 221 bp and *SsCTR1*: 461 bp) were PCR-amplified from the cDNA library of *S*. *sclerotiorum* wild-type strain by using the specific primers of RNAi vector construction in S3 Table. The sense and antisense fragments were ligated into the plasmid vector pCIT [57] at the corresponding sites, and a hygromycin resistance gene cassette from pSKH [58] was isolated and ligated into them, resulting the RNAi vectors, pSiatx, pSiccs and pSictr1. To generate *S*. *sclerotiorum* overexpression strains of these three genes, the full-length coding sequences were amplified by using the specific primers of overexpression vector construction in S3 Table and cloned into a hygromycin resistance containing vector, driven by the *S*. *sclerotiorum* constitutive expression promoter EF1-A (*SS1G_06124*, translation elongation factor 1 alpha), resulting the overexpression constructs, pOXSsATX1, pOXSsCCS and pOXSsCTR1. *S*. *sclerotiorum* transformations were conducted with a standard polyethylene glycol (PEG)-mediated transformation protocol [59].

The T-DNA mutants of *A*. *thaliana* (*AtCCS*: SALK_025986C, *AtCCH*: SALK_118605C, *AtMT2A*: SALK_021037C, and *AtDRT112*: SALK_135199C) were obtained from the Arabidopsis Biological Resource Center, Ohio State University, USA. The homozygous T-DNA insertion lines were confirmed with primers flanking the T-DNA insertions (S3 Table) and the left border primer LB1.3 (ATTTTGCCGATTTCGGAAC). To generate the overexpression lines, the coding sequences of these four genes were amplified from a cDNA library of *A*. *thaliana* Col-0 leaves. The amplicons were digested with *Xba* I and *Xho* I and ligated into the plant expression vector pBinGlyRed3, which contains a gene encoded red fluorescent protein (DsRed). The resulting vectors (pBinGlyRed-*AtCCS*, pBinGlyRed-*AtCCH*, pBinGlyRed-*AtMT2A* and pBinGlyRed-*AtDRT112*) were introduced into the *Agrobacterium tumefaciens* strain GV3101 by electroporation [60] and transformed into *A*. *thaliana* Col-0 by the floral dip method [61].

### RNA-seq and network analysis

In our previous study [25], the stems of resistant and susceptible *B*. *oleracea* plants in a F_2_ population which derived from the cross between a resistant *B*. *oleracea* genotype C01 (*B*. *incana*) and a susceptible *B*. *oleracea* genotype C41 (*B*. *oleracea* var. *alboglabra*) were inoculated with *S*. *sclerotiorum*, and lesions at 0, 12 and 24 hpi were collected for transcriptome sequencing. To analyze the dynamic changes during infection, here we analyzed the DEGs of *S*. *sclerotiorum* and *B*. *oleracea* by using the DESeq package [62].

To reveal the pathways associated with ‘copper ion homeostasis’, the RNA of infected leaves was sequenced from *A*. *thaliana* wild-type line, T-DNA mutants and overexpression lines during infection. Briefly, the detached leaves were inoculated with *S*. *sclerotiorum* wild-type strain, and RNA from the lesions was extracted with the RNAprep pure Plant Kit (DP 432, Tiangen Biotech (BEIJING) CO., LTD). The sequencing library was generated using the Illumina RNA Library Prep Kit (NEB, USA) following the manufacturer’s recommendation, and sequenced on an Illumina HiSeq 4000 platform with three biological replicates. After removing low-quality reads and those with adapter sequences, poly-N sequence from the raw data, the clean reads were screened and aligned to the reference genomes of *A*. *thaliana* (https://www.arabidopsis.org/download/index.jsp) and *S*. *sclerotiorum* (http://fungidb.org/common/downloads/Current_Release/Ssclerotiorum1980UF-70/) by using the TopHat program (http://ccb.jhu.edu/software/tophat/index.shtml) [63] with default parameters except that the Q value was set to 100. Gene expression was quantified using htseq-count 0.6.1p2 (https://htseq.readthedocs.io/). The raw counts were normalized by TMM normalization using the edgeR package [64] and the differential expression analysis was carried out using the DESeq package [62].

The threshold determining the significance of DEGs among multiple tests was set at a false discovery rate (*FDR*) ≤ 0.001 and |log_2_ ratio| ≥ 1. GO (Gene Ontology) and KEGG (Kyoto Encyclopedia of Genes and Genomes) enrichment analyses were performed with an FDR ≤ 0.05 as the threshold using AgriGO [65] and KOBAS 3.0 (http://kobas.cbi.pku.edu.cn/), respectively.

Weighted correlation networks were produced among the DEGs with R package WGCNA (Weighted Gene Co-expression Network Analysis) [66]. Networks were visualized by Cytoscape v3.4 [67].

### Measurement of copper concentration

In order to test copper flow in the leaves infected with *S*. *sclerotiorum*, we measured the copper concentration in hosts, including *B*. *napus* cv. Zhongshuang 11 with the moderate resistance [68], *B*. *napus* cv. Westar with high susceptibility, parental *B*. *oleracea* (C01 and C41) and *A*. *thaliana* lines. The detached leaves were inoculated with *S*. *sclerotiorum* strains, and the uninfected (U), margin (M) and necrotic (N) area of leaves were collected to measure area size by ImageJ (https://imagej.nih.gov/ij/). The tissues were washed with distilled water, dried for one week at 80°C, and then washed with 11 N HNO_3_. Copper content of tissues was measured using atomic absorption spectroscopy (SPECTR AA220) with flame at a wavelength of 324.8 nm. The copper concentration was determined with copper content per area (ug/cm^2^).

### Quantitative RT-PCR

Gene expression was analyzed by qRT-PCR using a Bio-Rad CFX96 Real Time System (Bio-Rad, USA) and QuantiTect SYBR Green PCR master mix (Bio-Rad, USA), according to the manufacturer’s instructions. The *SsTubulin* and *BoActin3* genes were used as the internal control for *S*. *sclerotiorum* and *B*. *oleracea*, respectively. All the qRT-PCR primers were listed in S3 Table. The PCR cycling conditions comprised 1 cycle of 95°C for 30 s, then 39 cycles of 95°C for 5 s and 55–70°C for 1 min, followed by a melting curve ramping from 65°C to 95°C with temperature increasing by 0.5°C every 5 s (1 cycle). Transcript levels were calculated from the threshold cycle using the 2^-ΔΔCT^ method [69]. Three replicates of same sample were performed for each gene and data were analyzed using CFX Manager v3.0.

### Pathogenicity assays

Pathogenicity of *S*. *sclerotiorum* was evaluated by infecting *B*. *napus* and *A*. *thaliana* according to the procedure described previously [25, 37]. The detached leaves at seedling stage of *B*. *napus* and *A*. *thaliana* were inoculated with mycelium-colonized agar plugs (0.6 cm for *B*. *napus*, 0.2 cm for *A*. *thaliana*) obtained from expanding margins of PDA-cultured colonies. The inoculation chamber was maintained at 85% relative humidity at 22°C. The lesion size (*S*, cm^2^) for the leaves was calculated with the formula *S* = π_*_a_*_b/4, where *a* and *b* represent the long and short diameter of an approximately elliptical lesion.

### Antioxidant activity assays

The antioxidant activity of *S*. *sclerotiorum* hyphae and *A*. *thaliana* leaves was determined by NBT staining [70]. Hyphae on the PDA plate at 24 hpi were infiltrated under gentle vacuum with NBT staining solution for 5 hours and then washed 3 times with distilled water, prior to observation with a microscope. *A*. *thaliana* leaves were infiltrated under gentle vacuum with NBT staining solution for 5 hours and then the staining solution was replaced with bleaching solution (ethanol: acetic acid: glycerol = 3:1:1). After 15 ± 5 min in a boiling water bath (~90–95°C), the bleaching solution was replaced with fresh bleaching solution and stained in 60% glycerin. The quantitative of ·O_2_^-^ accumulation was tested using the superoxide anion assay kit (A052-1-1; Nanjing Jiancheng Bioengineering Institute) according to the manufacturer’s protocol. The enzyme activity of Cu/Zn SOD of *S*. *sclerotiorum* hyphae and infected *A*. *thaliana* leaves was tested using a Cu/Zn SOD assay kit (A001-4-1; Nanjing Jiancheng Bioengineering Institute) according to the manufacturer’s protocol.

## Supporting information

S1 Fig DEG (differentially expressed gene) analysis of *Sclerotinia sclerotiorum* and *Brassica oleracea* (24 hours post inoculation [hpi] vs 12 hpi). (A) DEGs of *S*. *sclerotiorum* during infection in the resistant (R-Ss) and susceptible (S-Ss) *B*. *oleracea*. (B) DEGs of resistant (R-Bol) and susceptible (S-Bol) *B*. *oleracea*. (C) Heat map of *S*. *sclerotiorum* DEGs involved in the process ‘copper ion import’ and ‘copper ion transport’. (D) Heat map of *B*. *oleracea* DEGs involved in the process ‘copper ion homeostasis’. Ss R24/R12: the *S*. *sclerotiorum* DEGs in resistant *B*. *oleracea* by comparing 24 hpi to 12 hpi; Ss S24/S12: the *S*. *sclerotiorum* DEGs in susceptible *B*. *oleracea* by comparing 24 hpi to 12 hpi; Bol R24/R12: the *B*. *oleracea* DEGs in resistant *B*. *oleracea* by comparing 24 hpi to 12 hpi; Bol S24/S12: the *B*. *oleracea* DEGs in susceptible *B*. *oleracea* by comparing 24 hpi to 12 hpi.
(TIF)

S2 Fig Expression changes in genes of interest during infection. (A) Expression changes as determined by RNA-Seq (black bars) and quantitative real-time reverse transcription-polymerase chain reaction (qRT-PCR) (grey bars) for eight *S*. *sclerotiorum* genes involved in the processes of ‘copper ion import’ and ‘copper ion transport’. (B) Expression changes as estimated by RNA-Seq (black bars) and qRT-PCR (grey bars) for ten *B*. *oleracea* genes in the biological process of ‘copper ion homeostasis’. (C) qRT-PCR analysis of seven *B*. *oleracea* genes in the biological process of ‘copper ion homeostasis’ in resistant (C01) and susceptible (C41) parental *B*. *oleracea* lines. Error bars indicate the standard deviation of three independent samples. The quantity of *SsTubulin* and *BoActin3* cDNA normalized different samples in *S*. *sclerotiorum* and *B*. *oleracea*, respectively.
(TIF)

S3 Fig Features of the overexpression lines and T-DNA mutants of ‘copper ion homeostasis’ genes of *A*. *thaliana*. (A) Gene structure of homologs in *A*. *thaliana* (*AtCCS*, *AtMT2A*, *AtDRT112* and *AtCCH*) involved in the ‘copper ion homeostasis’. (B) Construction of the overexpression (OX) vectors of *A*. *thaliana* genes. (C) Relative expression level of the target genes in the overexpression and T-DNA *A*. *thaliana* lines as determined by qRT-PCR. The quantity of *A*. *thaliana AtActin8* cDNA normalized different samples. Error bars indicate the standard deviation of three independent samples. *: represents significant difference from the wild-type line at the level of 0.05 (Student's *t*-test).
(TIF)

S4 Fig Analysis of *A*. *thaliana* DEGs during infection. (A) Relative expression level of target genes in the *A*. *thaliana* T-DNA mutants and overexpression lines (OX) in comparison with the wild-type line as revealed by the RNA-seq. (B) GO terms (overlapped among the four genes) significantly enriched among the up-regulated DEGs at 12 hpi between *A*. *thaliana* overexpression (OX) lines and wild-type line (WT) and between OX and T-DNA mutants.
(TIF)

S5 Fig DEG network analysis of *A*. *thaliana* and *S*. *sclerotiorum* during infection. (A) Weighted Gene Co-expression Network Analysis (WGCNA) of the DEGs between overexpression lines (OX) and T-DNA mutants in *A*. *thaliana*. (B) GO terms significantly enriched among 394 DEGs in the red module in (A). The network was visualized using Cytoscape v3.4. (C) WGCNA of *S*. *sclerotiorum* DEGs during infection of *A*. *thaliana* overexpression lines and T-DNA mutants.
(TIF)

S6 Fig Construction and virulence assays of the wild-type strain, silenced and overexpression strains of *S*. *sclerotiorum*. (A) The silenced (RNAi: RNA interference) and overexpression (OX) vectors. (B) Relative expression level of the target genes in silenced, overexpression and wild-type strain 1980 on PDA medium as determined by qRT-PCR. The quantity of *S*. *sclerotiorum SsTubulin* cDNA normalized different samples. Error bars indicate the standard deviation of three independent samples. *: represents significant difference from wild-type strain at the level of 0.05 (Student's *t*-test). (C) Disease in *A*. *thaliana* wild-type seedlings infected with wild-type strain at 4 dpi (days-post inoculation). (D) Lesion size of *A*. *thaliana* wild-type line, mutants and overexpression lines inoculating with wild-type strain, the silenced and overexpression strains of three *S*. *sclerotiorum* genes at 24 hpi.
(TIF)

S7 Fig The growth on PDA for wild-type strain, silenced and overexpression strains with supplementing low and high-concentration of CuSO_4_. Error bars indicate standard deviation of five independent replicates with five plates for every sample in one replicate. *: represents significant difference from the wild-type strain at the level of 0.05 (Student's *t*-test).
(TIF)

S8 Fig Antioxidant activity of *A*. *thaliana* mutants and overexpression lines infected with different *S*. *sclerotiorum* strains. Quantitative of ·O_2_^-^ accumulation in *A*. *thaliana* leaves infected by wild-type strain, the silenced and overexpression strains of three *S*. *sclerotiorum* genes at 12 hpi. Error bars indicate standard deviation of five independent replicates.
(TIF)

S9 Fig Expression of *S*. *sclerotiorum* copper detoxification genes during infection. The quantity of *S*. *sclerotiorum SsTubulin* cDNA normalized different samples. Error bars indicate the standard deviation of three independent samples.
(TIF)

S1 Table 7321 *A*. *thaliana* DEGs between the overexpression lines and T-DNA mutants. (XLSX)

S2 Table 1273 *S*. *sclerotiorum* DEGs during infecting *A*. *thaliana* overexpression lines and T-DNA mutants. (XLSX)

S3 Table Primers used in this study. (XLSX)
