# Supplementary figures and images for: Sclerotinia sclerotiorum utilizes host-derived copper for ROS detoxification and infection
Source: PLoS Pathog. 2020 Oct 1;16(10):e1008919. doi: 10.1371/journal.ppat.1008919 (PMC7553324; doi:10.1371/journal.ppat.1008919)

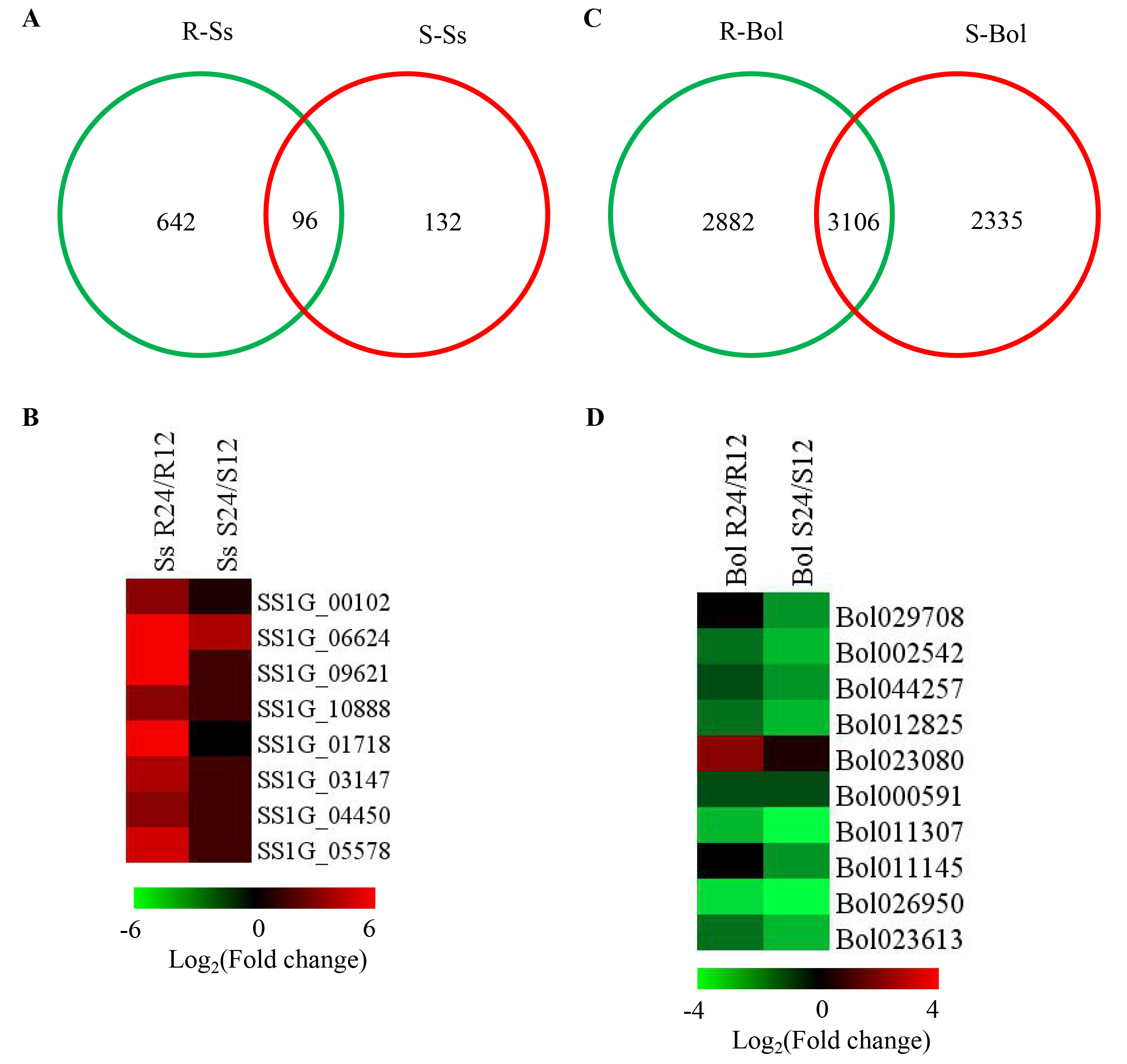

Supplement: S1 Fig — (A) DEGs of S. sclerotiorum during infection in the resistant (R-Ss) and susceptible (S-Ss) B. oleracea. (B) DEGs of resistant (R-Bol) and susceptible (S-Bol) B. oleracea. (C) Heat map of S. sclerotiorum DEGs involved in the process ‘copper ion import’ and ‘copper ion transport’. (D) Heat map of B. oleracea DEGs involved in the process ‘copper ion homeostasis’. Ss R24/R12: the S. sclerotiorum DEGs in resistant B. oleracea by comparing 24 hpi to 12 hpi; Ss S24/S12: the S. sclerotiorum DEGs in susceptible B. oleracea by comparing 24 hpi to 12 hpi; Bol R24/R12: the B. oleracea DEGs in resistant B. oleracea by comparing 24 hpi to 12 hpi; Bol S24/S12: the B. oleracea DEGs in susceptible B. oleracea by comparing 24 hpi to 12 hpi. (TIF) [file ppat.1008919.s001.tif]

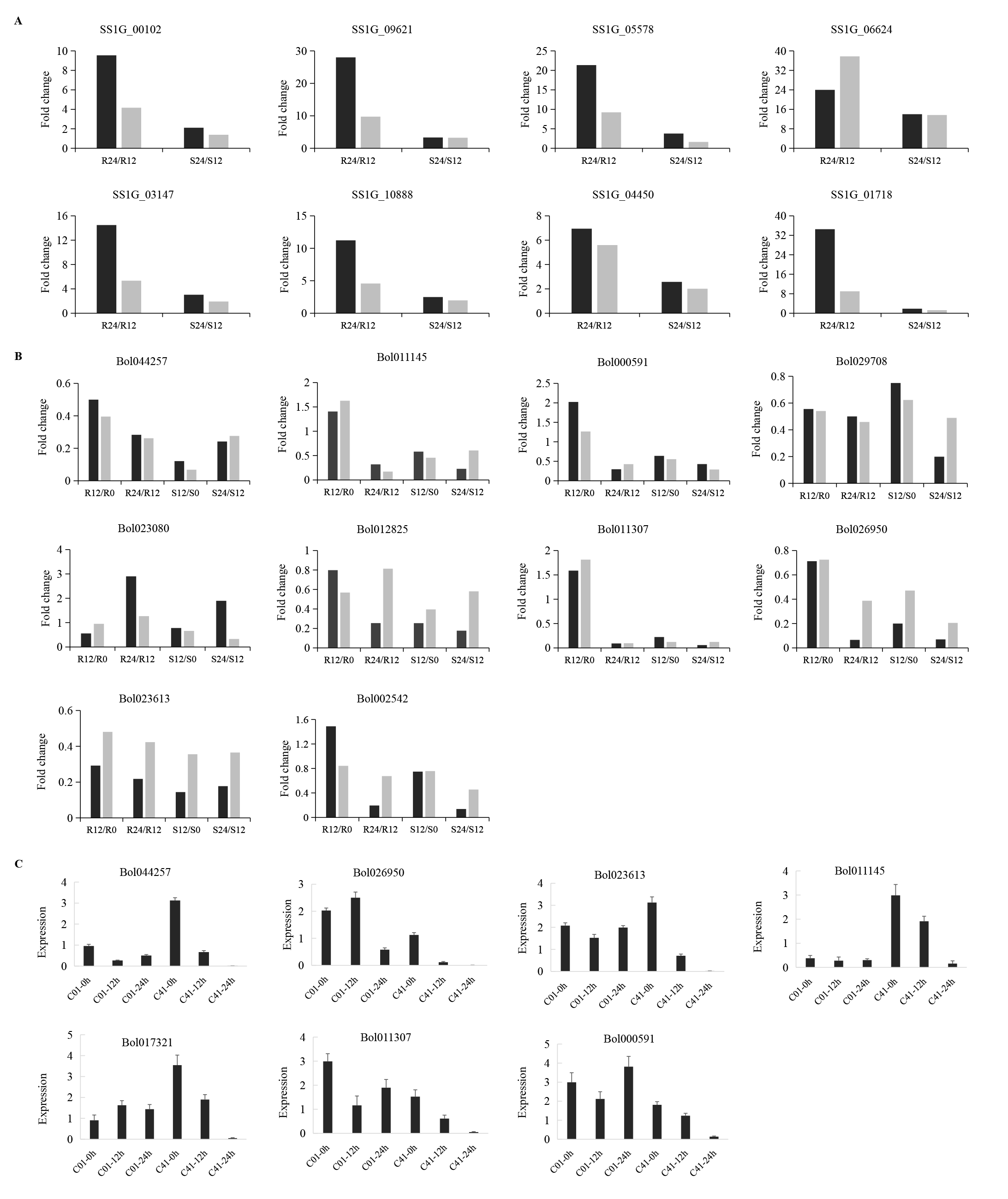

Supplement: S2 Fig — (A) Expression changes as determined by RNA-Seq (black bars) and quantitative real-time reverse transcription-polymerase chain reaction (qRT-PCR) (grey bars) for eight S. sclerotiorum genes involved in the processes of ‘copper ion import’ and ‘copper ion transport’. (B) Expression changes as estimated by RNA-Seq (black bars) and qRT-PCR (grey bars) for ten B. oleracea genes in the biological process of ‘copper ion homeostasis’. (C) qRT-PCR analysis of seven B. oleracea genes in the biological process of ‘copper ion homeostasis’ in resistant (C01) and susceptible (C41) parental B. oleracea lines. Error bars indicate the standard deviation of three independent samples. The quantity of SsTubulin and BoActin3 cDNA normalized different samples in S. sclerotiorum and B. oleracea, respectively. (TIF) [file ppat.1008919.s002.tif]

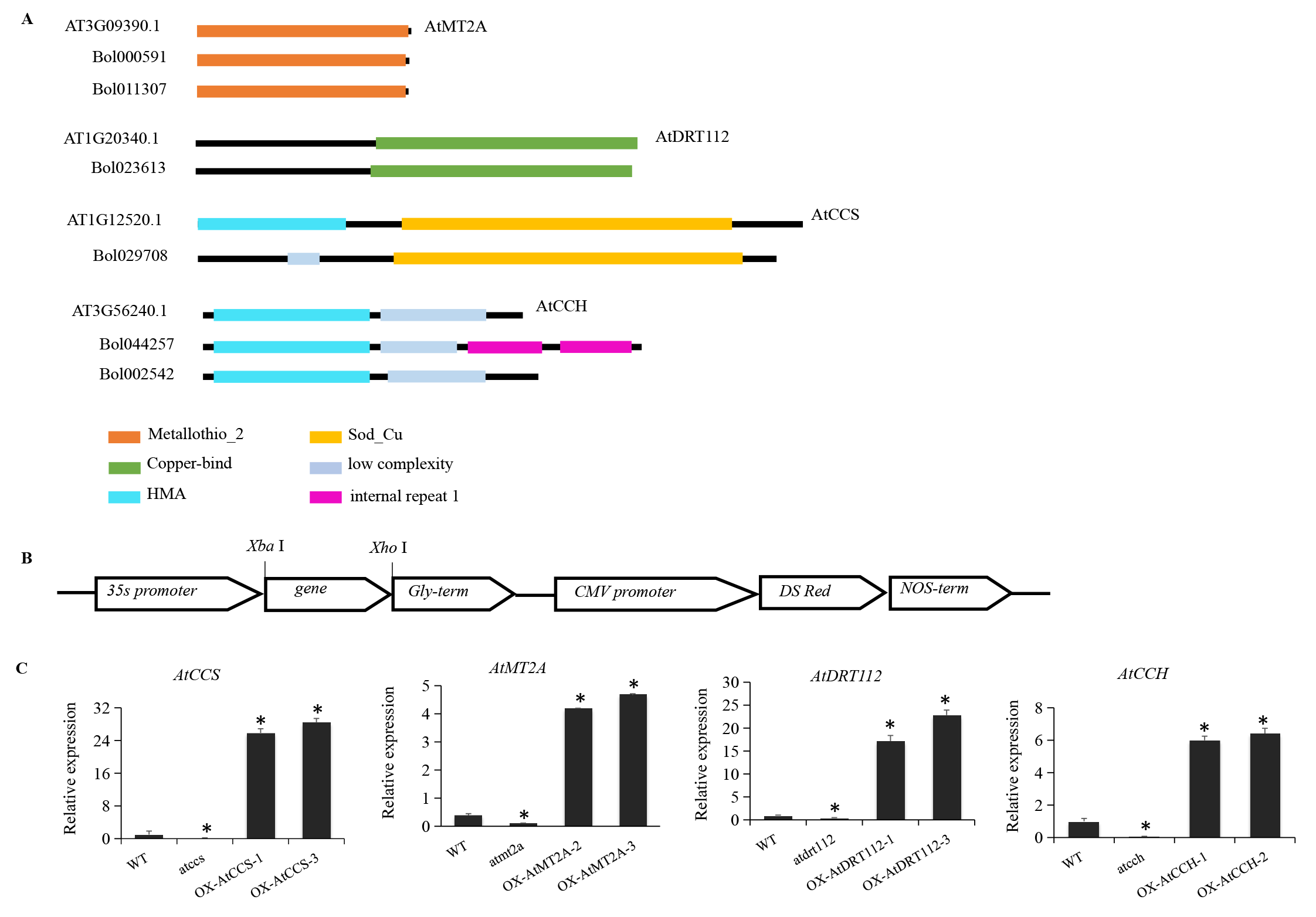

Supplement: S3 Fig — (A) Gene structure of homologs in A. thaliana (AtCCS, AtMT2A, AtDRT112 and AtCCH) involved in the ‘copper ion homeostasis’. (B) Construction of the overexpression (OX) vectors of A. thaliana genes. (C) Relative expression level of the target genes in the overexpression and T-DNA A. thaliana lines as determined by qRT-PCR. The quantity of A. thaliana AtActin8 cDNA normalized different samples. Error bars indicate the standard deviation of three independent samples. *: represents significant difference from the wild-type line at the level of 0.05 (Student's t-test). (TIF) [file ppat.1008919.s003.tif]

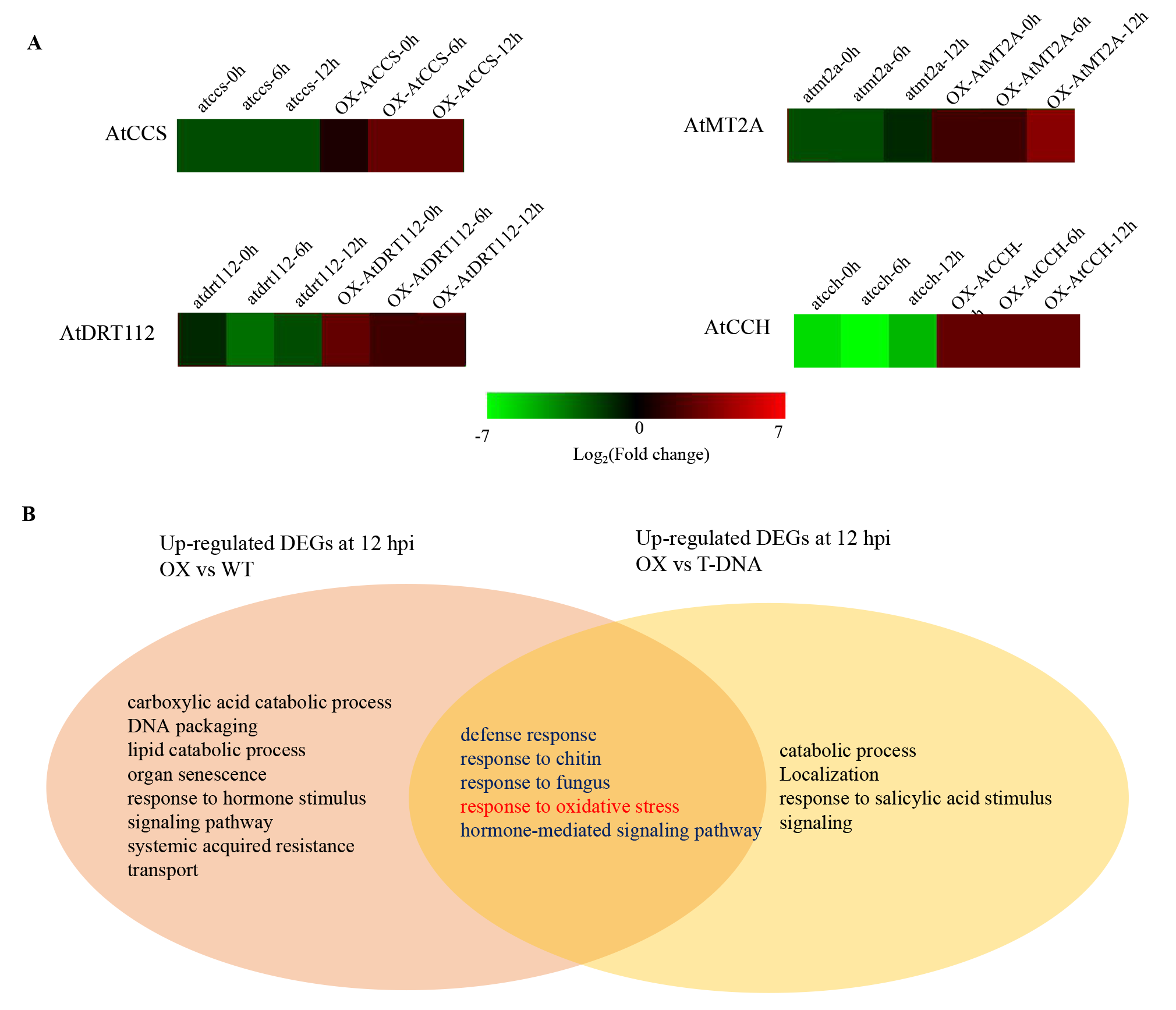

Supplement: S4 Fig — (A) Relative expression level of target genes in the A. thaliana T-DNA mutants and overexpression lines (OX) in comparison with the wild-type line as revealed by the RNA-seq. (B) GO terms (overlapped among the four genes) significantly enriched among the up-regulated DEGs at 12 hpi between A. thaliana overexpression (OX) lines and wild-type line (WT) and between OX and T-DNA mutants. (TIF) [file ppat.1008919.s004.tif]

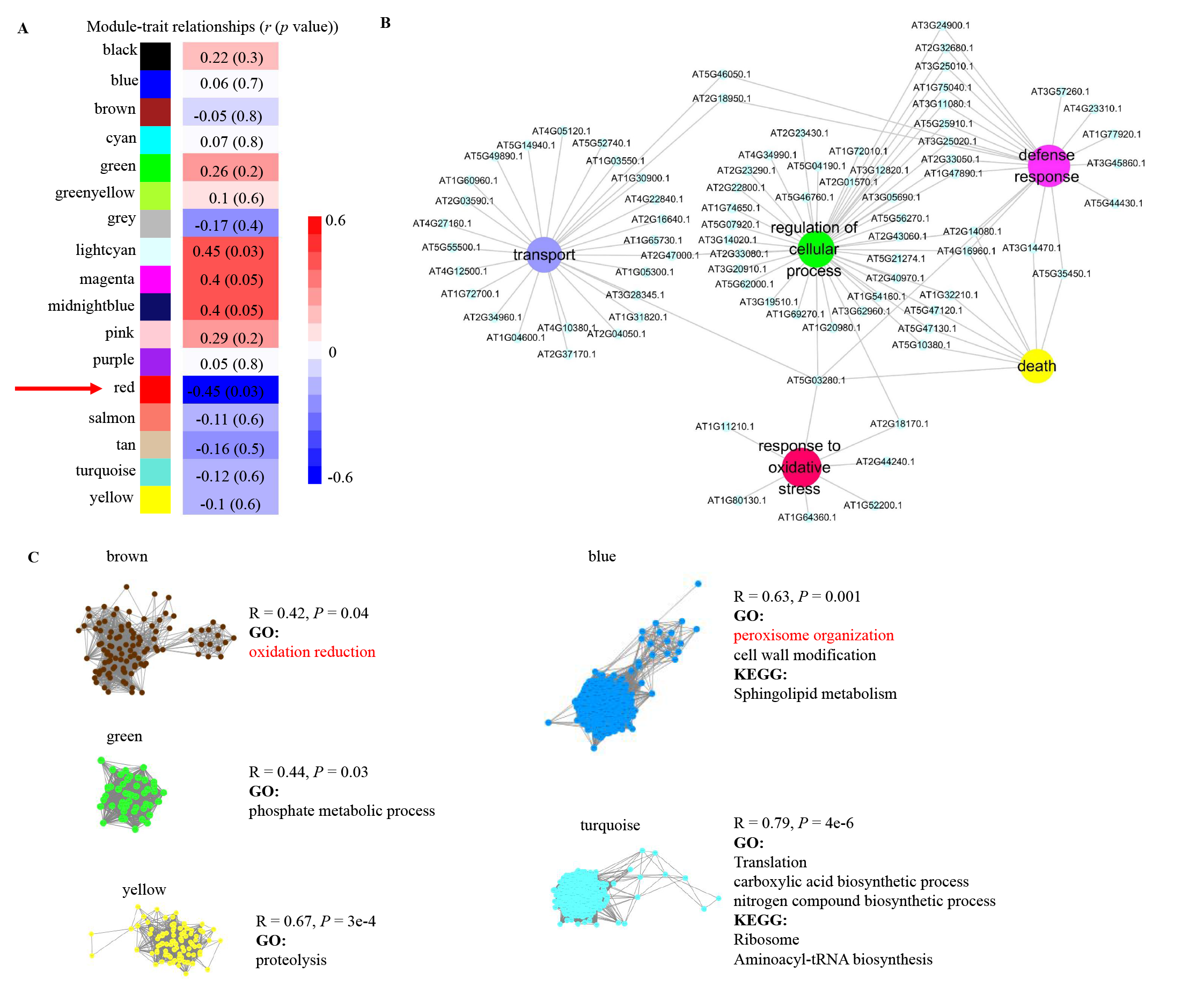

Supplement: S5 Fig — (A) Weighted Gene Co-expression Network Analysis (WGCNA) of the DEGs between overexpression lines (OX) and T-DNA mutants in A. thaliana. (B) GO terms significantly enriched among 394 DEGs in the red module in (A). The network was visualized using Cytoscape v3.4. (C) WGCNA of S. sclerotiorum DEGs during infection of A. thaliana overexpression lines and T-DNA mutants. (TIF) [file ppat.1008919.s005.tif]

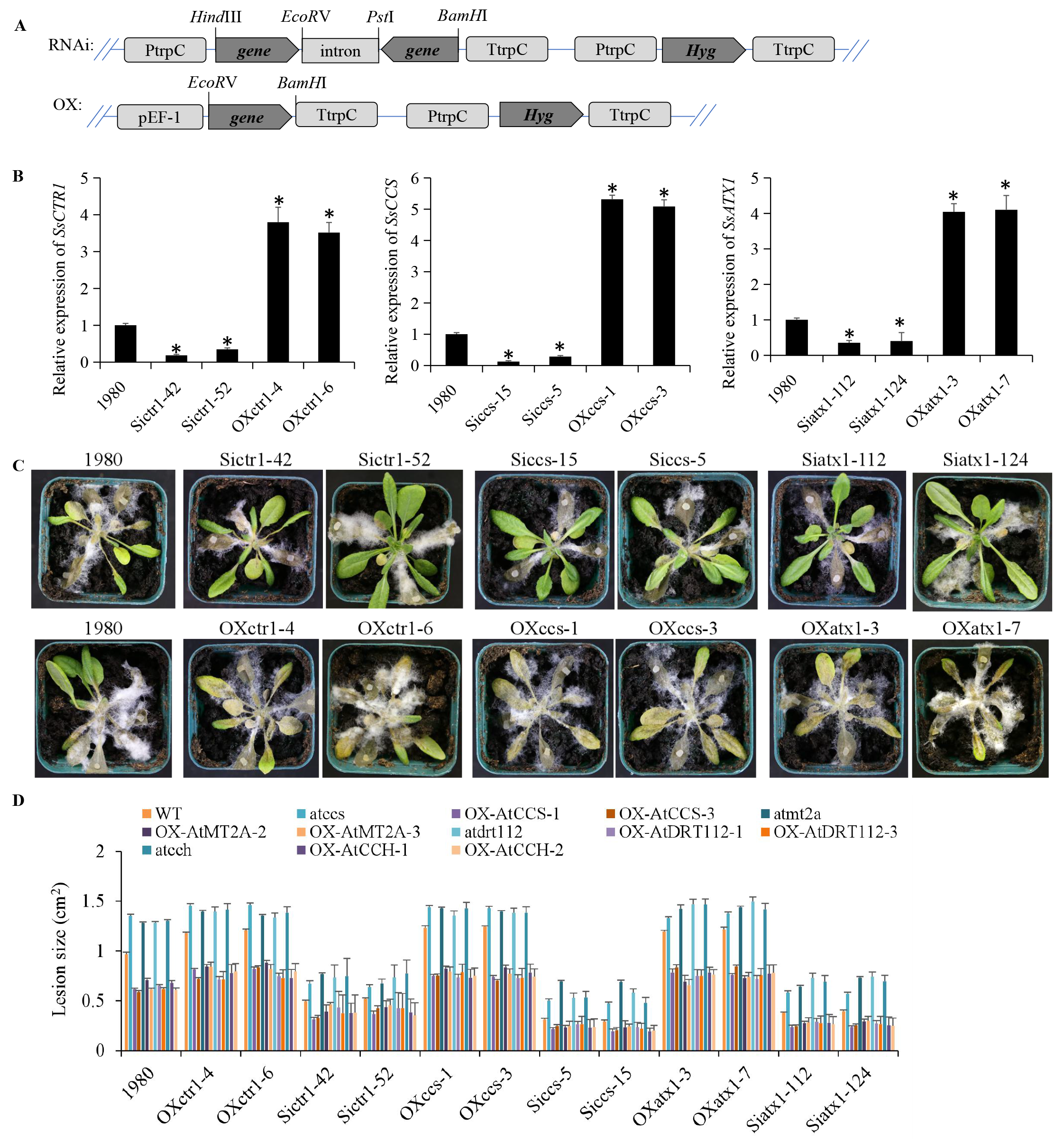

Supplement: S6 Fig — (A) The silenced (RNAi: RNA interference) and overexpression (OX) vectors. (B) Relative expression level of the target genes in silenced, overexpression and wild-type strain 1980 on PDA medium as determined by qRT-PCR. The quantity of S. sclerotiorum SsTubulin cDNA normalized different samples. Error bars indicate the standard deviation of three independent samples. *: represents significant difference from wild-type strain at the level of 0.05 (Student's t-test). (C) Disease in A. thaliana wild-type seedlings infected with wild-type strain at 4 dpi (days-post inoculation). (D) Lesion size of A. thaliana wild-type line, mutants and overexpression lines inoculating with wild-type strain, the silenced and overexpression strains of three S. sclerotiorum genes at 24 hpi. (TIF) [file ppat.1008919.s006.tif]

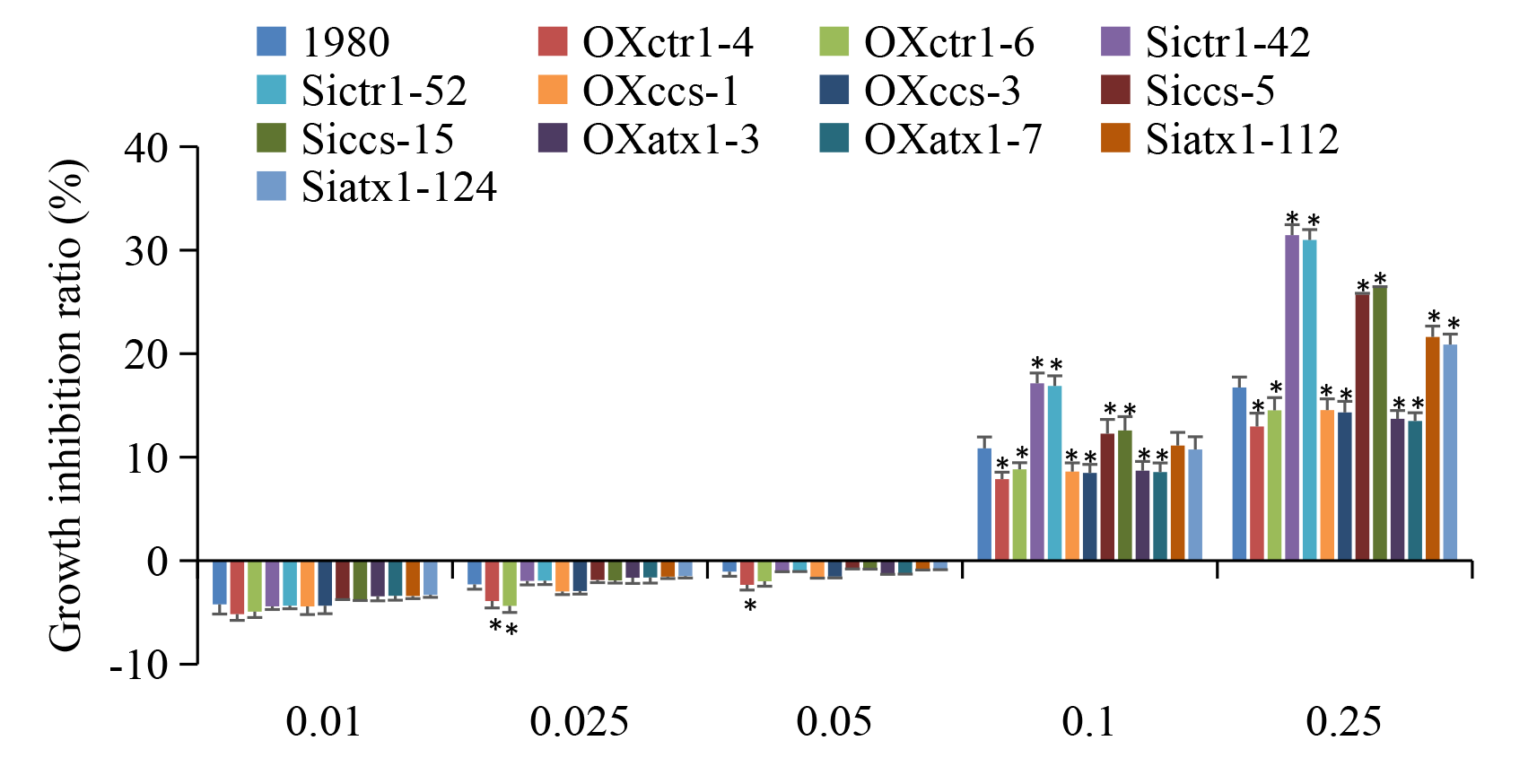

Supplement: S7 Fig — Error bars indicate standard deviation of five independent replicates with five plates for every sample in one replicate. *: represents significant difference from the wild-type strain at the level of 0.05 (Student's t-test). (TIF) [file ppat.1008919.s007.tif]

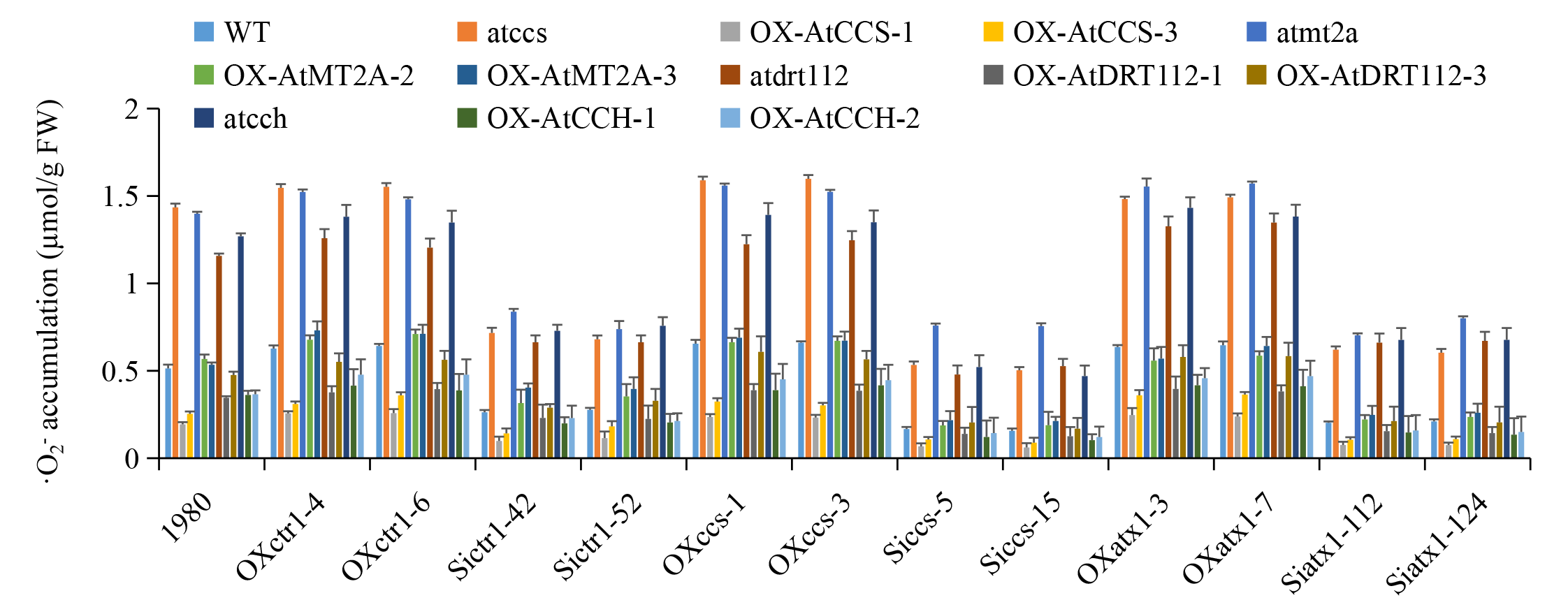

Supplement: S8 Fig — Quantitative of ·O2- accumulation in A. thaliana leaves infected by wild-type strain, the silenced and overexpression strains of three S. sclerotiorum genes at 12 hpi. Error bars indicate standard deviation of five independent replicates. (TIF) [file ppat.1008919.s008.tif]

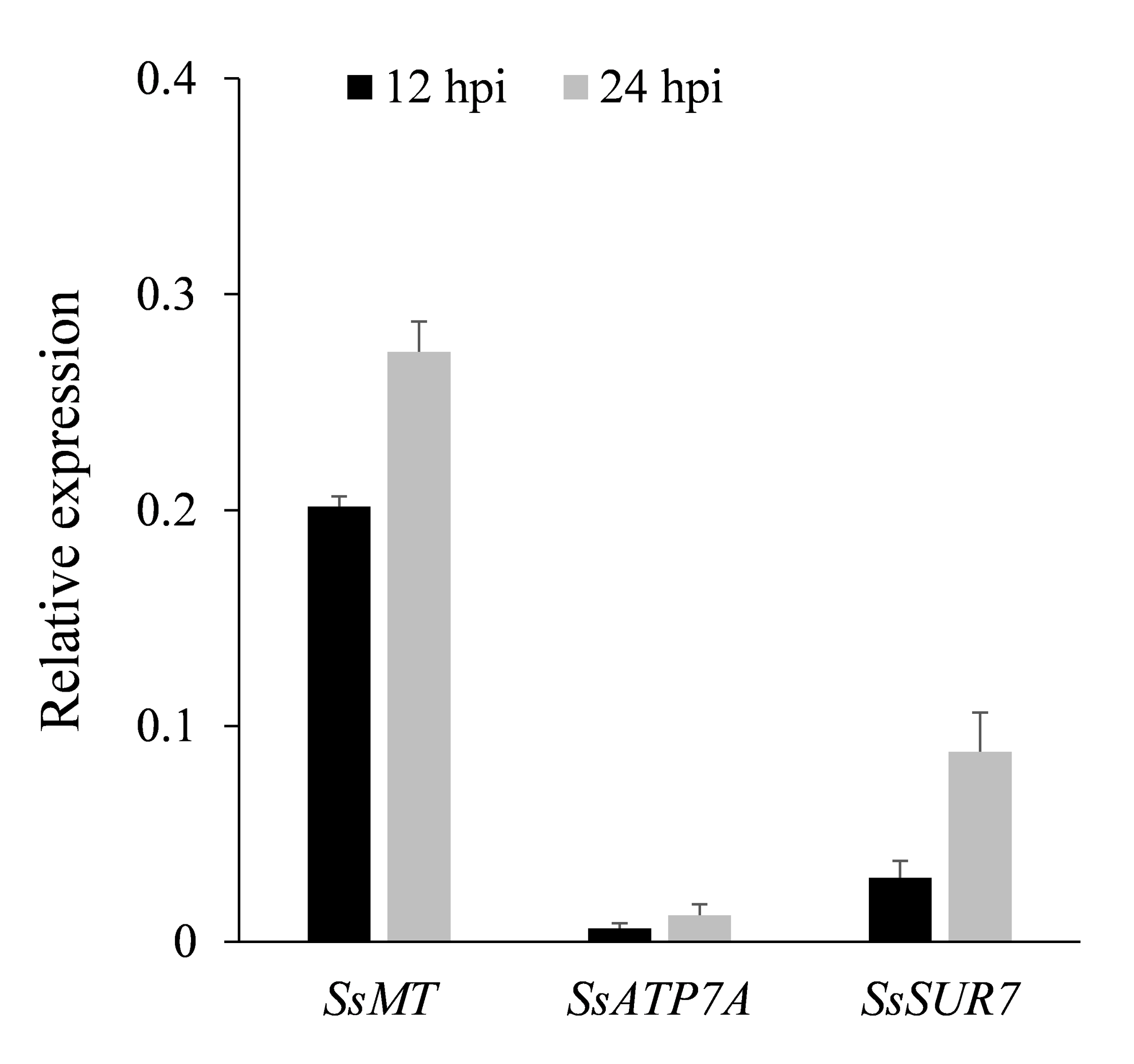

Supplement: S9 Fig — The quantity of S. sclerotiorum SsTubulin cDNA normalized different samples. Error bars indicate the standard deviation of three independent samples. (TIF) [file ppat.1008919.s009.tif]
